# A multi-centre open-label two-arm randomised superiority clinical trial of azithromycin versus usual care in ambulatory COVID-19: study protocol for the ATOMIC2 trial

**DOI:** 10.1186/s13063-020-04593-8

**Published:** 2020-08-17

**Authors:** Timothy S. C. Hinks, Vicki S. Barber, Joanna Black, Susan J. Dutton, Maisha Jabeen, James Melhorn, Najib M Rahman, Duncan Richards, Daniel Lasserson, Ian D. Pavord, Mona Bafadhel

**Affiliations:** 1 Respiratory Medicine Unit and National Institute for Health Research (NIHR) Oxford Biomedical Research Centre (BRC), Nuffield Department of Medicine Experimental Medicine, University of Oxford, Oxfordshire, OX3 9DU UK; 2 Oxford Clinical Trials Research Unit, Nuffield Department of Orthopaedics, Rheumatology and Musculoskeletal Sciences, Botnar Research Centre, University of Oxford, Oxford, UK; 3 University Hospital Llandough, Cardiff, CF64 2XX UK; 4 Nuffield Department of Medicine, Oxford University, Oxford, UK; 5 Warwick Medical School, University of Warwick, Coventry, CV4 7AL UK

**Keywords:** COVID-19, Coronavirus, SAR-CoV-2, Azithromycin, Macrolide, Randomised controlled trial, Respiratory failure, Mortality, Trial

## Abstract

**Background:**

Azithromycin is an orally active synthetic macrolide antibiotic with a wide range of anti-bacterial, anti-inflammatory and antiviral properties. It is a safe, inexpensive, generic licenced drug available worldwide and manufactured to scale and is a potential candidate therapy for pandemic coronavirus disease 2019 (COVID-19). Azithromycin was widely used to treat severe SARS-CoV and MERS-CoV, but to date, no randomised data are available in any coronavirus infections.

Other ongoing trials are exploring short courses of azithromycin either in early disease, within the first 7 days of symptoms, when azithromycin’s antiviral properties may be important, or late in disease when anti-bacterial properties may reduce the risk of secondary bacterial infection. However, the molecule’s anti-inflammatory properties, including suppression of pulmonary macrophage-derived pro-inflammatory cytokines such as interleukins-1β, -6, -8, and -18 and cytokines G-CSF and GM-CSF may provide a distinct therapeutic benefit if given in as a prolonged course during the period of progression from moderate to severe disease.

**Methods:**

ATOMIC2 is a phase II/III, multi-centre, prospective, open-label, two-arm randomised superiority clinical trial of azithromycin versus standard care for adults presenting to hospital with COVID-19 symptoms who are not admitted at initial presentation. We will enrol adults, ≥ 18 years of age assessed in acute hospitals in the UK with clinical diagnosis of COVID-19 infection where management on an ambulatory care pathway is deemed appropriate. Participants will be randomised in a 1:1 ratio to usual care or to azithromycin 500 mg orally daily for 14 days with telephone follow-up at days 14 and 28. The primary objective is to compare the proportion with either death or respiratory failure requiring invasive or non-invasive mechanical ventilation over 28 days from randomisation. Secondary objectives include mortality/respiratory failure in those with a PCR-confirmed diagnosis; all-cause mortality; progression to pneumonia; progression to severe pneumonia; peak severity of illness and mechanistic analysis of blood and nasal biomarkers.

**Discussion:**

This trial will determine the clinical utility of azithromycin in patients with moderately severe, clinically diagnosed COVID-19 and could be rapidly applicable worldwide.

**Trial registration:**

ClinicalTrials.gov NCT04381962. Registered on 11 May 2020. EudraCT identifier 2020-001740-26. Opened for accrual on 29 May 2020.

## Background

Azithromycin (AZM) is an orally active synthetic macrolide antibiotic with a wide range of antibacterial, anti-inflammatory and antiviral properties. It is a safe, inexpensive, generic licenced drug available worldwide, on the World Health Organization (WHO) list of essential medications, and manufactured to scale and therefore an ideal candidate molecule to be repurposed as a potential candidate therapy for pandemic COVID-19. Macrolides, particularly azithromycin, were used to treat 1/3 of severe cases of MERS-CoV [1], and azithromycin has been tried in COVID-19 infection [2] although RCT data for any coronavirus disease are lacking [3].

### Antiviral properties

Azithromycin has well-documented, broad antiviral properties in vitro. Numerous studies have shown it to be effective against respiratory viruses, including the picornavirus human rhinovirus (RV), where it enhances viral-induced type I and type III interferons and interferon-stimulated gene (ISG) expression and reduced RV replication and release [4–6]. Macrolides reduce RV replication in vitro by enhancing type I and III IFN and induce the antiviral ISGs viperin and MxA [6]. In vivo in a large, well-designed, RCT of 420 adults with severe asthma, long-term AZM strikingly reduced exacerbations by 40% over 1 year [7]. These effects occurred irrespective of inflammatory phenotype, and may be mediated by antiviral effects, as viruses trigger up to 80% of exacerbations in asthma [8, 9].

AZM has antiviral activity against SARS-CoV-2 in vitro*,* being shown to reduce viral replication in combination with hydroxychloroquine [10]. Azithromycin was also associated with a reduced viral load of non-SARS-CoV-2 alpha- and betacoronaviruses in children receiving AZM during a mass distribution programme [11]. In a separate drug screen of 1520 approved drugs, AZM was also a key hit with an EC_50_ of 2.1 μM [12]. Macrolides have also shown efficacy in vitro against a wide range of other viruses. These include the flavivirus Zika, where AZM was a key hit in a drug screen of 2177 compounds and markedly reduce viral proliferation and virus-induced cytopathic effects [13]. In Zika, AZM upregulates type 1 and type III interferon responses and the viral pathogen recognition receptors MDA5 and RIG-I and increases the levels of phosphorylated TBK1 and IRF3 [14]. There is also evidence of in vitro activity against enteroviruses [15], Ebola [16, 17] and SARS [18], with in vivo activity against influenza A, with reduction in IL-6, IL-8, IL-17, CXCL9, sTNF and CRP in a small open-label RCT [19].

### Anti-inflammatory properties

It is likely that AZM’s anti-inflammatory properties—rather than antiviral—will be more important in the treatment of severe COVID-19 disease in secondary care. Antivirals are likely to have limited efficacy in severe disease as they are administered late in the disease, after viraemia has peaked [20–22]. In stark contrast to the early cytokine storm responsible for 50% of deaths from influenza A, most COVID-19-related deaths occur due to sudden, late respiratory decompensation, peaking at day 14 after the onset of symptoms [23]. By this time, viral loads are low, and it is during the adaptive immunity stage that a late increase of innate/acute phase inflammatory cytokines occurs, including IL-1β, IL-2, IL-6, IL-7, IL-8, GCSF, MCP, MIP1a and TNF [24], and is associated with poor outcome [24]. These dysregulated cytokines are associated with features of haemophagocytic lymphohistiocytosis [25] and interstitial mononuclear inflammatory infiltrates, dominated by lymphocytes [26]. This points to a failure not of viral control, but of the ability to halt an over-exuberant inflammatory cascade. Therefore, the priority should be to target the off switch for these signalling cascades, which are characteristically steroid-resistant [22] and associated with pulmonary inflammation and extensive lung damage in SARS patients [27] and MERS-CoV [24, 28].

AZM’s anti-inflammatory properties include dose-dependent suppression of lymphocyte expression of perforin and of many of these cytokines, including IL-1β, IL-6 and TNF, IL-8(CXCL8), IL-18, G-CSF and GM-CSF [29–32] and other components of the IL-1β/IL-6-induced acute phase response such as serum amyloid protein A [30]. For these reasons, they have proven clinical efficacy in asthma, COPD, CF and obliterative bronchiolitis, post lung transplant obliterative bronchiolitis and diffuse pan bronchiolitis (DPB): a disease characterised by alveolar accumulation of foamy macrophages [29, 33]. In DPB, macrolide therapy has dramatically increased survival from 10–20% to 90% [29, 34, 35], attributed to AZM’s inhibition of dysregulated IL-1, IL-2, TNF and GM-CSF [36].

A key cell in the steroid-resistant ARDS which develops in COVID-19 are pro-inflammatory monocyte-derived macrophages [37], which are increased in severe disease, replacing alveolar macrophages [38]. Macrophage-derived cytokines tend to be resistant to corticosteroids. It is also a cell type markedly impaired by diabetes, a dominant risk factor for COVID-19-related death. An important property of macrolides is that they accumulate 100–1000-fold [29, 30] in lysosomes of phagocytes and are released in those sites when they die. Within the alveolar macrophage, AZM attenuates LPS-induced expression of pro-inflammatory cytokines through inhibition of AP-1 [39, 40]; it inhibits arachidonic acid release in LPS-stimulated macrophages [41], inhibits GM-CSF [30, 39, 42] and increases phagocytosis, likely by upregulation of CD206, the macrophage mannose receptor [43]. AZM attenuates type 1 response and shifts macrophage polarisation to a more immunosuppressive, tissue repair M2-phenotype [44–46]. Thus, AZM reduces M1 macrophage markers CCR7, CXCL11, IL-12p70 and enhanced IL-10 and CCL18.

### Anti-bacterial properties

Whilst not the main rationale for its use in COVID-19, the broad antibacterial properties of AZM which is active against a range of gram-positive, gram-negative, anaerobic and atypical infections may reduce secondary infection which were found in 16% of COVID-19 deaths [23].

### Justification for dose regimen

AZM is generally well-tolerated with a very good and well-documented safety record. It is associated with diarrhoea. Whilst there have been concerns about cardiovascular risk, huge epidemiological studies suggest these are very small effects (e.g. 47 extra deaths/million prescriptions) or perhaps have no effect when corrected for confounding. It is contraindicated in known hypersensitivity to the drug. It can be used in pregnancy. It should be used in caution in those receiving some other drugs including fluoroquinolones such as moxifloxacin and levofloxacin and in patients with ongoing proarrhythmic conditions.

Due to its long half-life, AZM accumulates over time, but to achieve a rapid effect, we will use 500 mg OD for 14 days, similar to the dose recommended in UK for treatment of Lyme disease [47]. This dose is selected to be known to be well-tolerated and of sufficient duration to cover the period during which progression from moderate to severe disease may occur and during which anti-inflammatory effects may be most beneficial.

### Rationale for design

This trial is designed to determine whether azithromycin is effective in preventing progression to severe respiratory failure requiring ventilatory support or death in adult patients with clinically diagnosed COVID-19 infection being assessed in secondary care but initially managed on an ambulatory care pathway. This specific situation provides a therapeutic window of opportunity to avert development of more severe disease.

## Methods

### Objectives

#### Aim

The aim is to test the hypothesis that the use of azithromycin 500 mg once daily for 14 days is effective in preventing and/or reducing the severity of lower respiratory illness of COVID-19 disease at 28 days.

#### Primary objective

The primary objective is to compare the effect of azithromycin in participants with a clinical diagnosis of COVID-19 in reducing the proportion with either death or hospital admission with respiratory failure requiring invasive or non-invasive mechanical ventilation over 28 days from randomisation.

#### Secondary objectives

The secondary objectives are to compare the effect of azithromycin in participants with a PCR-confirmed diagnosis of COVID-19 in reducing the proportion with either death or hospital admission with respiratory failure requiring invasive or non-invasive mechanical ventilatory support over 28 days from randomisation (for those who had a COVID-19 swab at randomisation), to compare differences in all-cause mortality, to compare differences in proportion progressing to pneumonia, to compare differences in proportion progressing to severe pneumonia and to compare differences in peak severity of illness.

#### Exploratory objectives

The exploratory objective is the mechanistic analysis of blood and nasal biomarkers if available.

### Trial design

**Fig. 1:**
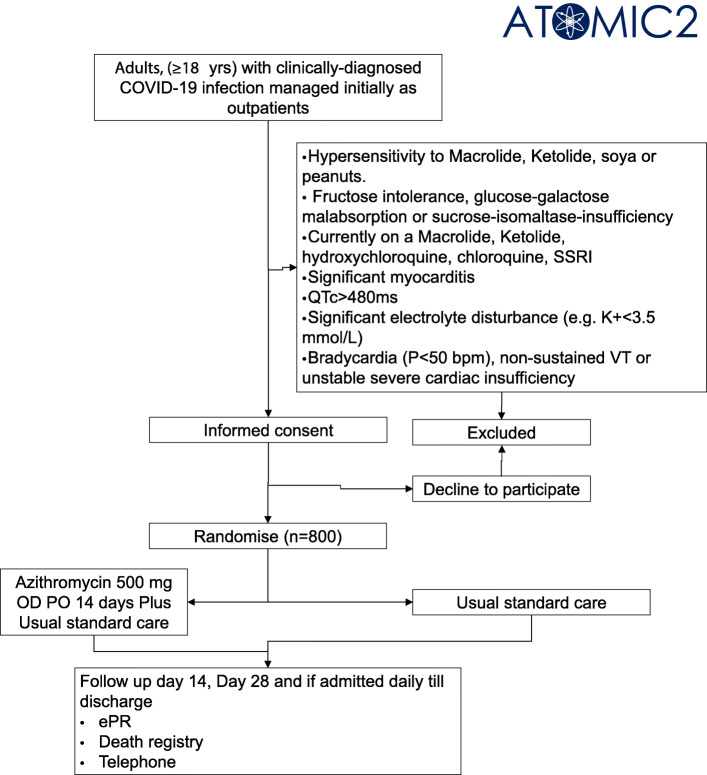
CONSORT (Consolidated Standards of Reporting Trials) flow diagram

This is a phase II/III, multi-centre, prospective open-label two-arm randomised superiority clinical trial of standard care versus azithromycin with standard care alone for those presenting to hospital with COVID-19 symptoms who are not admitted at initial presentation (Fig. 1). The study procedures are outlined in Table 1.

**Table 1:** SPIRIT (Standard Protocol Items: Recommendations for Interventional Trials) Schedule of Events Timeline: enrolment, assessment of safety, assessment of efficacy and dispensing of the medicine

	Study day			
Procedures	Day 0 (day of randomisation)	14 days after randomisation (study day 14)—participant contacted by phone	28 days after randomisation (study day 28)—participant contacted by phone	Any hospital admission
Consent	✓			
Eligibility check	✓			
Demographics	✓			
Medical history	✓			
Medication history	✓	✓	✓	✓
Swab taken for COVID PCR test (if possible)	✓			
Randomisation	✓			
Dispensing of 14 day course of IMP (if randomised to IMP)	✓			
Medical notes/ePR/biochemistry results/microbiology results review	✓			✓
Radiology review (if any performed on clinical grounds)	✓			✓
Assessment of outcome measures (vital status, history of admission) (ePR/notes/death register/telephone call)		✓	✓	✓
Compliance assessment (telephone call)		✓	✓	
Study blood sampling (optional)	✓ (serum sample + Tempus, EDTA)			✓
Nasal brush (optional, for observational)	✓			
SAE/AE reporting	✓	✓	✓	✓

## Discussion

The current COVID-19 pandemic has galvanised an unprecedented response within the global research community. AZM has been rapidly identified by a number of researchers as of potential utility based on theoretical grounds [48] and on data from in vitro drug screening [12] of molecules which could be repositioned. This approach of repositioning existing drugs has the advantage of known safety profiles and that drugs can be rapidly produced at relatively low cost and so being quicker to deploy than a new molecule or vaccine. AZM in particular is already synthesised at scale globally by a number of manufacturers and so is particularly attractive for repurposing.

The SARS-CoV-2 pandemic is now the third novel coronavirus to emerge in the last two decades, each with pandemic potential, and the emergence of further novel coronaviruses is to be expected. Despite widespread use in SARS-CoV and MERS-CoV, no randomised data yet exist. Therefore, well-designed randomised trials are urgently required. If efficacy is proven in such studies, then there is urgency in discovering, communicating and translating these findings. Equally, if robust randomised trials find convincing evidence of no therapeutic benefit, then this is also essential information to disseminate. Azithromycin is an extremely useful drug for a wide variety of indications including treatment or prevention of a range of bacterial infections of the respiratory tract, ear, skin and soft tissue, genital tract, and eye [47], as well as having proven efficacy against mycobacteria and inflammatory lung conditions [29, 34, 35]. Unfortunately, AZM is particularly prone to induction of antimicrobial resistance, with high levels of resistance already occurring globally [49], making restriction of unnecessary use a clear priority.

Several other trials of AZM in COVID-19 have been initiated including two national studies in UK [50, 51]. However, given the strong rationale for investigating this molecule, multiple trials are needed. If one trial provides evidence of clinical efficacy in one particular population or clinical setting, then further studies will be needed to provide data on which other populations and settings and dose regimens are appropriate. These trials differ in several significant aspects. Firstly, they are studying different time points in the disease course: for instance, the PRINCIPLE trial is investigating people within the first 7 days of symptoms, the RECOVERY trial in late disease after hospitalisation with severe clinical features. Secondly, they are recruiting in different settings, the former in primary care, the latter in secondary care. Thirdly, they are using different dose regimens, the former just 3 days of therapy, the latter 5 days. Together, these studies are therefore likely to be assessing different properties of AZM: in early disease, PRINCIPLE will explore antiviral effects, in very late disease RECOVERY is likely to expose anti-bacterial activities against secondary infection, whilst ATOMIC2 is intermediate and predominantly assessing AZM’s anti-inflammatory effects.

ATOMIC2 is positioned between primary and secondary care populations with several unique features. Selection of a population who have presented to secondary care will focus recruitment on those with significant early symptoms and a high risk (20–30%) of readmission within the next 2 weeks, and yet, we believe, not too late in the disease process for suppression of pulmonary macrophage-derived inflammatory cytokines to have a potentially clinically meaningful beneficial effect.

Azithromycin is safe and well tolerated. Even in long-term administration (500 mg thrice weekly for 48 weeks), there was no increase in serious adverse events besides mild increase in diarrhoea [7]. The main adverse event of concern in this trial would be potential cardiovascular toxicity. Although macrolides have a class warning for potential cardiac QT prolongation, azithromycin does not show this effect under experimental conditions [52]. Only a few cases of QT prolongation have been reported for patients treated with the drug [53], mainly because azithromycin, unlike other macrolide antibiotics, does not interact with CYP3A4, despite a minor interaction with the anti-coagulant warfarin [54]. In the large AMAZES RCT, there was no increase in QTc prolongation, although this study excluded participants with QTc>480 ms [7]. Recently, a large study of Medicaid prescriptions reported an additional risk of cardiovascular death of 47 extra deaths/million compared with amoxicillin (relative risk (RR) for cardiovascular death 2.49 [55], and a meta-analysis of 20 million patients suggested a RR for cardiac death or ventricular tachycardia of 2.42 [56]. However, these effects are very small and subject to confounding and at odds with more recent studies: in a review of 185,000 Medicare patients, odds ratio for CV death was only 1.35, and after controlling for covariates decreased to 1.01 (0.95–1.08) [57], whilst a large Cochrane review of 183 trials found no evidence of an increase in cardiac disorders with macrolides (OR 0.87) [58]. Overall, the risk to a patient treated would be low compared with the considerable mortality of COVID-19, particularly if patients with QTc>480 ms were excluded.

Besides answering the question of clinical efficacy, this study will also provide valuable samples at baseline and at subsequent admission for both blood and nasal epithelial samples. Using proteomics, direct ex vivo functional T cell analyses and RNA studies, this will provide information both on potential biomarkers of response and also insight into the immune consequences of SARS-CoV-2 infection on the peripheral blood and airway epithelial cell transcriptome. Such insights into the pathogenic mechanisms of coronaviruses may prove valuable in directing research into future epidemics of this challenging family of emergent RNA viruses.

## Trial status

The trial commenced recruitment on 3 June 2020 according to protocol version 3.0 (7 May 2020), with adoption of protocol version 5.0 on 29 July 2020, with an anticipated completion date of December 2020.

### Trial registration

ATOMIC2 was registered with ClinicalTrials.gov NCT04381962 on 11 May 2020, EudraCT identifier 2020-001740-26, and opened for accrual on 29 May 2020.

### Full protocol

The full protocol (V3.0) is attached as an additional file, accessible from the Trials website (Additional file 1). V5.0 of the protocol was implemented on 16 July 2020 including significant chagnes to inclusion / exclusion criteria and is also attached (Additional file 2). In the interest in expediting dissemination of this material, the familiar formatting has been eliminated; this letter serves as a summary of the key elements of the full protocol. The study protocol has been reported in accordance with the Standard Protocol Items: Recommendations for Clinical Interventional Trials (SPIRIT) guidelines (Additional file 3). Patient Information Sheet (Additional file 4) and Informed Consent Form (Additional file 5) are attached as additional files.

## Supplementary information

**Additional file 1.**

**Additional file 2.**

**Additional file 3.**

**Additional file 4.**

**Additional file 5.**

## Data Availability

The research team will have access to the final trial dataset which will be hosted by the Oxford Clinical Trial Research Unit.

## Abbreviations

AZM: Azithromycin
BTS: British Thoracic Society
COVID-19: Coronavirus disease 2019
CURB-65: Confusion, urea > 7.0 mmol/L, respiratory rate ≥ 30 breaths/min, blood pressure < 90 systolic or ≤ 60 diastolic, age ≥ 65 years
EDTA: Ethylenediaminetetraacetic acid
ePR: Electronic patient record
IMV: Invasive mechanical ventilation
MERS: Middle East respiratory syndrome
NHS: National Health Service (UK)
PCR: Polymerase chain reaction
RNA: Ribonucleic acid
RV: Rhinovirus
SARS: Severe acute respiratory syndrome
SPIRIT: Standard Protocol Items: Recommendations for Interventional Trials
SSRI: Selective serotonin reuptake inhibitor
SST: Serum separator tube
QTc: Corrected QT interval
