# Supplementary material for: A multi-centre open-label two-arm randomised superiority clinical trial of azithromycin versus usual care in ambulatory COVID-19: study protocol for the ATOMIC2 trial
Source: Trials. 2020 Aug 17;21:718. doi: 10.1186/s13063-020-04593-8 (PMC7429453; doi:10.1186/s13063-020-04593-8)
Supplement: Supplementary file 1 — Additional file 1. [file 13063_2020_4593_MOESM1_ESM.pdf]

ATOMIC2

Trial Title: A multi-centre open-label two-arm randomised superiority clinical trial of Azithromycin versus usual care In Ambulatory COVID-19 (ATOMIC2)
Internal Reference Number / Short title: ATOMIC2

IRAS Project ID: 282892 **REC Ref:** 20/HRA/2105

EudraCT Number: 2020-001740-26

Date and Version No: 07May2020, V3.0

Chief Investigator: Dr Timothy SC Hinks, University of Oxford

Investigators: Prof Mona Bafadhel, University of Oxford

Dr Maisha Jabeen, University of Oxford / Buckinghamshire Healthcare NHS Trust

Prof Daniel Lasserson, City Hospital Birmingham, Sandwell and West Birmingham Hospitals NHS Trust

Prof Ian Pavord, University of Oxford

Prof Duncan Richards, University of Oxford

Sponsor: University of Oxford, Clinical Trials and Research Governance, Joint Research Office, Joint Research Office, Boundary Brook House, Headington, Oxford, OX3 7GB ctrg@admin.ox.ac.uk

Funder: Oxford NIHR Biomedical Research Centre Respiratory Theme and University of Oxford MSD COVID-19 Research Response Fund and Pfizer Inc.

Additional funding applications pending.

Chief Investigator Signature:

Statistician Signature:

I declare the team have no relevant financial conflicts of interest

Confidentiality Statement

This document contains confidential information that must not be disclosed to anyone other than the Sponsor, the Investigator Team, HRA, host organisation, and members of the Research Ethics Committee and Regulatory Authorities unless authorised to do so.

© Copyright: The University of Oxford and Oxford University Hospitals NHS Foundation Trust 2019

TABLE OF CONTENTS

Trial Title: A multi-centre open-label two-arm randomised superiority clinical trial of Azithromycin versus usual care In Ambulatory COVID-19 (ATOMIC2) 1

1 KEY TRIAL CONTACTS 6

2 LAY SUMMARY 7

3 SYNOPSIS 8

4 ABBREVIATIONS 11

5 BACKGROUND AND RATIONALE 13

5.1 Antiviral properties 13

5.2 Anti-inflammatory properties 14

5.3 Antibacterial properties 15

5.4 Justification for using Azithromycin and dose regimen 15

5.5 Rationale for design 15

5.6 Potential additional study arms 16

6 OBJECTIVES AND OUTCOME MEASURES 16

6.1 Hypothesis 16

7 TRIAL DESIGN 19

8 PARTICIPANT IDENTIFICATION 21

8.1 Trial Participants 21

8.2 Inclusion Criteria 21

8.3 Exclusion Criteria 22

9 TRIAL PROCEDURES 22

9.1 Recruitment 22

9.2 Screening and Eligibility Assessment 22

9.3 Informed Consent 23

9.4 Randomisation 23

9.5 Blinding and code-breaking 24

9.6 Baseline Assessments (Day 0) 24

9.6.1 Severity scale score for peak severity of illness 25

9.7 Subsequent assessments 26

9.7.1 At study recruitment (optional for all sites and participants) 27

9.7.2 Sample handling, processing and analysis 27

© Copyright: The University of Oxford and Oxford University Hospitals NHS Foundation Trust 2019

9.8 GP notification 28
9.9 Early Discontinuation/Withdrawal of Participants 28
9.10 Definition of End of Trial 29
10 SAFETY REPORTING 29
10.1 Adverse Event Definitions 29
10.2 Reportable Events for ATOMIC2 30
10.3 Assessment of Causality 30
10.4 Procedures for Reporting Adverse Events 31
10.5 Reporting Procedures for Serious Adverse Events 31
10.5.1 Events exempt from reporting as SAEs 31
10.5.2 Procedure for immediate reporting of Serious Adverse Events 32
10.6 Expectedness 32
10.7 SUSAR Reporting 32
10.8 Development Safety Update Reports 32
11 TRIAL INTERVENTIONS 33
11.1 Investigational Medicinal Product(s) (IMP) Description 33
11.1.1 Study intervention 33
11.1.2 Dose regimen 33
11.1.3 Authorisation and safety 33
11.1.4 Contraindications 34
11.1.5 Cautions 34
11.1.6 Pregnancy 35
11.1.7 Blinding of IMPs 35
11.1.8 Storage and dispensing of IMP 35
11.1.9 Compliance with Trial Treatment 36
11.1.10 Concomitant Medication 36
11.1.11 Post-trial treatment 36
11.2 Other Treatments (non-IMPs) 36
11.3 Other Interventions 36
12 STATISTICS 36
12.1 Statistical Analysis Plan (SAP) 36
12.2 Description of Statistical Methods 37

© Copyright: The University of Oxford and Oxford University Hospitals NHS Foundation Trust 2019

12.3 Sample Size Determination ... 37
12.4 Analysis Populations ... 38
12.5 Stopping Rules ... 38
12.6 The Level of Statistical Significance ... 39
12.7 Procedure for Accounting for Missing, Unused, and Spurious Data. ... 39
12.8 Procedures for Reporting any Deviation(s) from the Original Statistical Plan ... 39
13 DATA MANAGEMENT ... 39
13.1 Source Data ... 39
13.2 Access to Data ... 39
13.3 Data Recording and Record Keeping ... 40
13.4 Collection of data ... 40
14 QUALITY ASSURANCE PROCEDURES ... 41
14.1 Risk assessment ... 41
14.2 Monitoring ... 41
14.3 Quality assurance ... 41
14.4 Trial committees ... 41
14.4.1 Data Safety Monitoring Committee (DSMC) ... 41
14.4.2 Trial Steering Committee (TSC) ... 42
15 PROTOCOL DEVIATIONS ... 42
16 SERIOUS BREACHES ... 42
17 ETHICAL AND REGULATORY CONSIDERATIONS ... 43
17.1 Declaration of Helsinki ... 43
17.2 Guidelines for Good Clinical Practice ... 43
17.3 Approvals ... 43
17.4 Reporting ... 43
17.5 Transparency in Research ... 43
17.6 Participant Confidentiality ... 43
17.7 CTU Involvement ... 44
18 FINANCE AND INSURANCE ... 44
18.1 Funding ... 44
18.2 Insurance ... 45
18.3 Contractual arrangements ... 45

© Copyright: The University of Oxford and Oxford University Hospitals NHS Foundation Trust 2019

19 PUBLICATION POLICY 45

20 DEVELOPMENT OF A NEW PRODUCT/ PROCESS OR THE GENERATION OF INTELLECTUAL PROPERTY 45

21 ARCHIVING 45

22 REFERENCES 46

23 APPENDIX A: Figure 1 – Background data for trial rationale 50

24 APPENDIX B: SCHEDULE OF PROCEDURES 51

25 APPENDIX C: EXAMPLE OF PARTICIPANT’S STUDY JOURNEY 52

26 APPENDIX D: AMENDMENT HISTORY 53

© Copyright: The University of Oxford and Oxford University Hospitals NHS Foundation Trust 2019

1 KEY TRIAL CONTACTS

**Chief Investigator**	Dr Timothy SC Hinks, timothy.hinks@ndm.ox.ac.uk<br>NDM, Level 5, John Radcliffe Hospital, Headley Way, OX3 9DU<br>T: +44 1865 220885<br>M: +44 771 2261970
**Sponsor**	University of Oxford<br>Clinical Trials and Research Governance, Joint Research Office, Joint Research Office, Boundary Brook House, Headington, Oxford, OX3 7GB, ctrg@admin.ox.ac.uk
**Funder(s)**	Funded by the National Institute for Health Research (NIHR) Oxford Biomedical Research Centre (BRC) Respiratory Theme & University of Oxford MSD COVID-19 Research Response Fund
**Clinical Trials Unit**	Oxford Clinical Trials Research Unit<br>University of Oxford<br>Botnar Research Centre<br>Windmill Road<br>Oxford<br>OX3 7LF<br>Tel: 01865 223469 atomic2@ndorms.ox.ac.uk
**Statisticians**	Susan Dutton and Ariel Wang<br>Oxford Clinical Trials Research Unit, Tel: 01865 223469
**Committees**	**Data safety monitoring committee (DSMC)**<br>Prof James Chalmers (Chair)<br>University of Dundee, Nethergate, Dundee, Scotland, UK, DD1 4HN j.chalmers@dundee.ac.uk<br><br>**Trial steering committee (TSC)**<br>Prof Paul Little (Chair)<br>University of Southampton; School of Primary Care, Population Sciences and Medical Education, Aldermoor Health Centre, Aldermoor Close, Southampton, SO16 5ST p.little@soton.ac.uk

© Copyright: The University of Oxford and Oxford University Hospitals NHS Foundation Trust 2019

2 LAY SUMMARY

Coronavirus-induced disease 2019 (COVID-19) is an infection caused by a virus whose full name is severe acute respiratory syndrome coronavirus 2 (SARS-CoV-2). This is a new and rapidly-spreading infectious disease. Those people that come into contact with the virus can have symptoms such as a mild fatigue, fever, loss of taste and a persistent cough, which can develop into severe respiratory failure requiring hospitalisation and mechanical ventilation. For those where the symptoms worsen this typically occurs 1 to 2 weeks into coming in contact with the virus. This provides a window of opportunity to potentially treat those patients who present with symptoms before becoming seriously ill to take a drug that might not result in them developing the severe symptoms. The ATOMIC2 study is investigating if a common antibiotic called Azithromycin (AZM) may prevent the patients from getting worse. Azithromycin is a safe, inexpensive, antibiotic that is available worldwide and is often prescribed by doctors across the world and it has been proved to have a wide range of antibacterial, anti-inflammatory and antiviral properties.

In this study, researchers want to investigate this medicine in patients who have mild symptoms of COVID-19 who go to the hospital, but who doctors decide there is no need to admit them for treatment. The study will investigate if half the patients are given Azithromycin for 14 days and half the patients do not receive Azithromycin, are there less people after 28 days in one of the groups who go on to develop more severe symptoms from COVID-19.

© Copyright: The University of Oxford and Oxford University Hospitals NHS Foundation Trust 2019

3 SYNOPSIS

Trial Title	A multi-centre open-label two-arm randomised superiority clinical trial of Azithromycin versus usual care In Ambulatory COVID-19 (ATOMIC2)		
Internal ref. no. (or short title)	ATOMIC2		
Trial registration	This trial will be registered with https://clinicaltrials.gov/		
Sponsor	University of Oxford<br>Clinical Trials and Research Governance, Joint Research Office, Joint Research Office, Boundary Brook House, Headington, Oxford, OX3 7GB, ctrg@admin.ox.ac.uk		
Funder	Funded by the National Institute for Health Research (NIHR) Oxford Biomedical Research Centre (BRC) Respiratory Theme and University of Oxford MSD COVID-19 Research Response Fund and Pfizer Inc		
Clinical Phase	Phase II/III		
Trial Design	Multi centre, prospective two-arm open-label randomized superiority clinical trial		
Trial Participants	Adults, ≥18 years of age with clinically-diagnosed COVID-19 infection managed initially as outpatients.		
Sample Size	Approximately 800 participants (400 per arm). The DSMC will review the accruing data, safety and efficacy data and a futility analysis once the first 100 participants have completed their 28 day follow-up. If progression to the full trial is recommended the endpoints and sample size assumptions will also be reviewed blinded to treatment allocation to refine the final definitive study sample size.		
Planned Trial Period	Treatment duration 14 days.<br>Duration of follow up: 28 days from randomisation unless admitted to hospital, when participant will be followed up until discharged.<br>Duration of study recruitment:<br>-Anticipated –up to 6 months		
Planned Recruitment period	May-December 2020		
	**Objectives**	**Outcome Measures**	**Timepoint(s)**
Primary	To compare the effect of Azithromycin in participants with a clinical diagnosis of COVID-19 in reducing the proportion with either death or hospital admission	Efficacy will be determined through differences in the proportion with either death or admission with respiratory failure requiring level 2 ventilation (NIV/CPAP/ nasal high flow) or level 3 IMV)	Day 28

© Copyright: The University of Oxford and Oxford University Hospitals NHS Foundation Trust 2019

	with respiratory failure requiring Non-Invasive Mechanical Ventilation (NIV) or Invasive Mechanical Ventilation (IMV) over the 28 days from randomisation.	over the 28 days from randomisation	
Secondary	To compare the effect of Azithromycin in participants with a PCR-confirmed diagnosis of COVID-19 in reducing the proportion with either death or hospital admission with respiratory failure requiring invasive or non-invasive mechanical ventilation over 28 days from randomisation.<br>(For those with SWAB results)	Efficacy will be determined through differences in the proportion with either death or admission with respiratory failure requiring level 2 ventilation (NIV/CPAP/ nasal high-flow) or level 3 (invasive mechanical ventilation) over 28 days from randomisation using a retrospective analysis of oropharyngeal swabs for those patients who had a COVID-19 swab obtained at time of randomisation.	Day 28
	To compare differences in all-cause mortality.	Data on vital status	Day 28
	To compare differences in pneumonia progression.	Progression to pneumonia. This will be diagnosed on a CXR or CT thorax with compatible clinical findings, where there was no consolidation on the baseline CXR.	Day 28
	To compare differences in proportion progressing to severe pneumonia.	Retrospective review of CXRs or CT thorax to determine evolution of pneumonia from pneumonia on baseline (CXR). Severe pneumonia is defined as BTS CURB-65 score 3-5.	Day 28

© Copyright: The University of Oxford and Oxford University Hospitals NHS Foundation Trust 2019

	To compare differences in peak severity of illness.	Maximum severity score during the study period will be recorded.	Ascertain from day 14 and 28 phone call and retrospective medical notes/ePR data at day 28 or death, whichever soonest.
	Safety and tolerability	Serious adverse events and concomitant medications. Record at enrolment, emergently during study period and proactively elicit at day 14 and at day 28.	Emergent data collection days 0-28 and elicit proactively at day 14 and day 28 post randomisation.
**Exploratory Objectives**	Mechanistic analysis of blood and nasal biomarkers if available	The following samples may be taken Blood for serum, Tempus tube (whole blood RNA), EDTA tubes (PBMC), nasal brush to be placed immediately into RNA lysis buffer (for subsequent PCR and transcriptomic analysis).	Samples to be collected prospectively at baseline and again if patient admitted, to be taken as soon as possible and within 72 hours of admission if possible.
Comparator	Standard care according to the hospital protocol where the patient is being triaged and there is a clinical decision not to admit the patient.		
Intervention IMP(s)	Azithromycin 500 mg orally daily for 14 days. This will be two 250mg capsules to be taken each day.		

© Copyright: The University of Oxford and Oxford University Hospitals NHS Foundation Trust 2019

4 ABBREVIATIONS

AE	Adverse event
ALT	Alanine aminotransferase
APTT	Activated partial thromboplastin time
AR	Adverse reaction
AST	Aspartate aminotransferase
AZM	Azithromycin
BP	Blood pressure
BTS	British Thoracic Society
CCL	Chemokine (C-C motif) ligand
CF	Cystic Fibrosis
CI	Chief Investigator
COPD	Chronic Obstructive Pulmonary Disease
CPAP	Continuous positive airway pressure (ventilation)
CRA	Clinical Research Associate (Monitor)
CRF	Case Report Form
CRP	C-reactive protein
CT	Computed tomogram
CTA	Clinical Trials Authorisation
CTRG	Clinical Trials and Research Governance
CURB-65	Confusion, Urea >7.0 mmol/L, Respiratory Rate >/=30 breaths/min, Blood pressure <90 systolic or </=60 diastolic, Age >/= 65 years.
CXR	Chest X-Ray
DMSC	Data Monitoring Committee / Data Monitoring and Safety Committee
DPB	Diffuse Pan Bronchiolitis
DSUR	Development Safety Update Report
EDTA	Ethylenediaminetetraacetic acid
ePR	Electronic patient record
FBC	Full blood count
GCP	Good Clinical Practice
GCSF	Granulocyte Colony Stimulating Factor
GP	General Practitioner

© Copyright: The University of Oxford and Oxford University Hospitals NHS Foundation Trust 2019

HFOT	High-Flow Oxygen Therapy: warmed, humidified oxygen delivered via a nasal mask at >15 L/min
HR	Heart Rate
HRA	Health Research Authority
ICF	Informed Consent Form
ICH	International Conference on Harmonisation
IL	Interleukin
IMP	Investigational Medicinal Product
IMV	Invasive mechanical ventilation (ventilatory support delivered via an endotracheal tube)
ISARIC	International Severe Acute Respiratory and Emerging Infection Consortium
ISG	Interferon Stimulated Gene
MHRA	Medicines and Healthcare products Regulatory Agency
MERS	Middle East Respiratory Syndrome
MMRM	Mixed Model for Repeated Measurement
MxA	A membrane protein
NIV	Non-invasive ventilation (ventilatory support via an external face mask in a non-sedated person)
NHS	National Health Service
PCR	Polymerase chain reaction
PEEP	Positive End-Expiratory Pressure
PI	Principal Investigator
PIL	Participant/ Patient Information Leaflet
PROBE	Prospective randomized open blinded end-point (PROBE) clinical trial
PT	Prothrombin time
QTc	Corrected QT interval
R&D	NHS Trust R&D Department
RCT	Randomised Controlled Trial
REC	Research Ethics Committee
RES	Research Ethics Service
RNA	Ribonucleic acid
RR	Respiratory Rate
RSI	Reference Safety Information

© Copyright: The University of Oxford and Oxford University Hospitals NHS Foundation Trust 2019

RV	RhinoVirus
SAE	Serious Adverse Event
SAR	Serious Adverse Reaction
SARS	Severe Acute Respiratory Syndrome
SDV	Source Data Verification
SMPC	Summary of Medicinal Product Characteristics
SOP	Standard Operating Procedure
SSRI	Selective Serotonin Reuptake Inhibitor
SST	Serum Separating Tube
SUSAR	Suspected Unexpected Serious Adverse Reactions
Tempus tube	Blood collection tubes for RNA purification
TMF	Trial Master File
U+E	Urea and electrolytes

5 BACKGROUND AND RATIONALE

Azithromycin (AZM) is an orally active synthetic macrolide antibiotic with a wide range of antibacterial, anti-inflammatory and antiviral properties. It is a safe, inexpensive, generic licensed drug available worldwide, on the WHO list of essential medications, and manufactured to scale and therefore an ideal candidate molecule to be repurposed as a potential candidate therapy for pandemic COVID-19. Macrolides, particularly Azithromycin, were used to treat 1/3 of severe cases of MERS-CoV[1] and Azithromycin has been tried in COVID-19 infection[2] although RCT data are lacking[3].

5.1 Antiviral properties

Azithromycin has well-documented, broad antiviral properties *in vitro*. Numerous studies have shown it to be effective against respiratory viruses, including the picornavirus human rhinovirus (RV), where it enhances viral-induced type I and type III interferons and interferon-stimulated gene (ISG) expression and reduced RV replication and release[4-6]. Macrolides reduce RV replication *in vitro* by enhancing type I and III IFN and induce the antiviral ISGs viperin and MxA[6] (Figure 1c, Appendix A). *In vivo* in a large, well-designed, RCT of 420 adults with severe asthma long term AZM strikingly reduced exacerbations by 40% over 1 year (Gibson, Lancet 2017)[7] (Figure 1a, Appendix A). These effects occurred irrespective of inflammatory phenotype, and are likely mediated by the antiviral effects, as viruses trigger 80% of exacerbations in asthma[8,9].

© Copyright: The University of Oxford and Oxford University Hospitals NHS Foundation Trust 2019

Macrolides have shown efficacy *in vitro* against a wide range of other viruses. These include the flavivirus Zika, where AZM was a key hit in a drug screen of 2177 compounds and markedly reduce viral proliferation and virus-induced cytopathic effects[10] (Figure 1b, Appendix A). In Zika, AZM upregulates type 1 and type III interferon responses and the viral pathogen recognition receptors MDA5 and RIG-I, and increases the levels of phosphorylated TBK1 and IRF3[11]. There is also evidence of *in vitro* activity against enteroviruses[12], Ebola[13,14] and SARS[15]; with *in vivo* activity against influenza A, with reduction in IL-6, IL-8, IL-17, CXCL9, sTNF and CRP in a small open label RCT[16] (Figure 1d, Appendix A).

5.2 Anti-inflammatory properties

It is likely that AZM's anti-inflammatory properties – rather than antiviral – will be more important in the treatment of severe COVID-19 disease in secondary care. Antivirals are likely to have limited efficacy in severe disease as they are administered late in the disease, after viraemia has peaked[17-19]. In stark contrast to the early cytokine storm responsible for 50% of deaths from influenza A, most COVID-19-related deaths occur due to sudden, late respiratory decompensation, peaking at day 14 after the onset of symptoms[20]. By this time viral loads are low, and it is during the adaptive immunity stage that a late increase of innate / acute phase inflammatory cytokines occurs, including IL-1β, IL-2, IL-6, IL-7, IL-8, GCSF, MCP, MIP1a, TNF[21] and associated with poor outcome[21]. These dysregulated cytokines are associated with features of hemophagocytic lymphohistiocytosis[22] and interstitial mononuclear inflammatory infiltrates, dominated by lymphocytes[23]. This points to a failure not of viral control, but of the ability to halt an over-exuberant inflammatory cascade. Therefore the priority should be to target the off-switch for these signalling cascades, which are characteristically steroid-resistant[19], and associated with pulmonary inflammation and extensive lung damage in SARS patients[24] and MERS-CoV[21,25].

AZM's anti-inflammatory properties include dose-dependent suppression of lymphocyte expression of perforin, and of many of these cytokines, including IL-1□, IL-6 and TNF, IL-8(CXCL8), IL-18, G-CSF and GM-CSF[26-29] and other components of the IL-1□/IL-6-induced acute phase response such as serum amyloid protein A[27]. For these reasons they have proven clinically efficacy in asthma, COPD, CF and obliterative bronchiolitis, post lung transplant obliterative bronchiolitis and diffuse pan bronchiolitis (DPB): a disease characterised by alveolar accumulation of foamy macrophages[26,30]. In DPB macrolide therapy has dramatically increased survival from 10-20% to 90%[26,31,32], attributed to AZM's inhibition of dysregulated IL-1, IL-2, TNF and GM-CSF[33].

A key cell in the steroid-resistant ARDS which develops in COVID-19 are pro-inflammatory monocyte-derived macrophages[34], which are increased in severe disease, replacing alveolar macrophages[35]. Macrophage-derived cytokines tend to be resistant to corticosteroids. It is also a cell type markedly impaired by diabetes, a dominant risk factor for COVID-19 related death. An important property of macrolides is that they accumulate 100-1000-fold[26,27] in lysosomes of

© Copyright: The University of Oxford and Oxford University Hospitals NHS Foundation Trust 2019

phagocytes and are released in those sites when they die. Within the alveolar macrophage AZM attenuates LPS-induced expression of pro-inflammatory cytokines through inhibition of AP-1[36,37], it inhibits arachidonic acid release in LPS-stimulated macrophages[38], inhibits GM-CSF[27,36,39] and increases phagocytosis, likely by upregulation of CD206, the macrophage mannose receptor[40]. AZM attenuates type 1 response and shifts macrophage polarization to a more immunosuppressive, tissue repair M2-phenotype[41-43]. Thus AZM reduces M1 macrophage markers CCR7, CXCL11, IL-12p70 and enhanced IL-10 and CCL18. This inhibitory profile is similar to that of chloroquine (another potential COVID-19 repurposed drug) due to their similar propensities for lysosomal accumulation.

5.3 Antibacterial properties

Whilst not the main rationale for its use in COVID-19, the broad antibacterial properties of AZM which is active against a range of gram positive, gram negative, anaerobic and atypical infections, may reduce secondary infection which were found in 16% of COVID-19 deaths[20], which could be sufficient grounds for a clinical trial irrespective of other effects, and therefore data on development of pneumonia will be analysed as a secondary outcome.

5.4 Justification for using Azithromycin and dose regimen

Azithromycin has marketing authorisation in the UK and in EU member states. AZM is generally well-tolerated with a very good and well-documented safety record. It is associated with diarrhoea. Whilst there have been concerns about cardiovascular risk, huge epidemiological studies suggest these are very small effects (e.g. 47 extra deaths / million prescriptions) or perhaps no effect when corrected for confounding. It is contraindicated in known hypersensitivity to the drug. It can be used in pregnancy. It should be used in caution in those receiving some other drugs including fluoroquinolones such as moxifloxacin and levofloxacin, and in patients with ongoing proarrhythmic conditions. [For full details see 'Investigational Medicinal Product Description' below and the Summary of Product Characteristics SmPC].

Due to its long half-life AZM accumulates over time, but to achieve a rapid effect we will use 500mg OD for 14 days, similar to the dose recommended for Lyme disease[44]

The trial will use commercial AZM provided by Pfizer. However, in the unlikely event of short supplies, the protocol will allow any authorised brand that contains the active ingredient azithromycin.

5.5 Rationale for design

We will therefore perform an efficacy study of AZM to prevent and/or reduce the severity of lower respiratory tract illness in adult patients with clinically-diagnosed COVID-19 infection being assessed in secondary care but initially managed on an ambulatory care pathway. This provides a

© Copyright: The University of Oxford and Oxford University Hospitals NHS Foundation Trust 2019

therapeutic window of opportunity to avert development of more severe disease. Participants will be randomised to receive Azithromycin 500 mg daily for 14 days or standard care. The first dose will be within 4 hours of randomisation.

5.6 Potential additional study arms

This area of research is rapidly evolving and it may become possible to include additional interventions as extra arms into this study to enable rapid assessment of potentially important treatments in this patient population. If this becomes possible a protocol amendment and amendments to associated documents (PIS, consent forms etc) will be submitted for approval to the sponsor, REC and Competent Authority before any changes are implemented and the comparison for any new intervention will use concurrent usual care controls.

6 OBJECTIVES AND OUTCOME MEASURES

6.1 Hypothesis

Use of Azithromycin 500 mg once daily for 14 days is effective in preventing and/or reducing the severity of lower respiratory illness of COVID-19 disease at 28 days.

Objectives	Outcome Measures	Time point(s) of evaluation of this outcome measure (if applicable)
**Primary Objective**<br>To compare the effect of Azithromycin in participants with a clinical diagnosis of COVID-19 in reducing the proportion with either death or hospital admission with respiratory failure requiring invasive or non-invasive mechanical ventilation over 28 days from randomisation.	Efficacy will be determined through differences in the proportion with either death or admission with respiratory failure requiring level 2 ventilation (NIV/CPAP/nasal high-flow) or level 3 (invasive mechanical ventilation) in the 28 days from randomisation.	Determined at day 28 from randomisation.

© Copyright: The University of Oxford and Oxford University Hospitals NHS Foundation Trust 2019

Objectives	Outcome Measures	Time point(s) of evaluation of this outcome measure (if applicable)
**Secondary Objectives**		
To compare the effect of Azithromycin in participants with a PCR-confirmed diagnosis of COVID-19 in reducing the proportion with either death or hospital admission with respiratory failure requiring invasive or non-invasive mechanical ventilatory support over 28 days from randomisation (for those who had a COVID-19 swab at randomisation)	Efficacy will be determined through differences in the proportion with either death or admission with respiratory failure requiring level 2 ventilatory support (NIV/CPAP/nasal high-flow) or level 3 (invasive mechanical ventilation) in the 28 days from randomisation using a retrospective analysis of COVID-19 oropharyngeal swabs for those who had one taken at time of randomisation.	Determined at day 28 from randomisation.
To compare differences in all-cause mortality.	Data on vital status (alive / dead, with date and presumed cause of death if appropriate) at 28 days from randomisation	Ascertain data at 28 days after randomisation.
To compare differences in proportion progressing to pneumonia.	Progression to pneumonia as diagnosed by chest x-ray (or CT thorax), with compatible clinical findings, if no pneumonia is present at time of enrolment. To be diagnosed by a medically qualified doctor and data obtained from review of case-notes and relevant radiology.	Ascertain this information at time of pneumonia diagnosis, or at 28 days after randomisation (whichever is sooner)
To compare differences in proportion progressing to severe pneumonia.	Evolution of pneumonia, as diagnosed by chest x-ray or CT thorax, if pneumonia is present at time of enrolment. To be diagnosed by a medically qualified doctor and data	Ascertain this information retrospectively at

© Copyright: The University of Oxford and Oxford University Hospitals NHS Foundation Trust 2019

**Objectives**	**Outcome Measures**	**Time point(s) of evaluation of this outcome measure (if applicable)**
	obtained from review of case-notes and relevant radiology. Severe pneumonia is defined as BTS CURB-65 score of 3-5.	28 days after randomisation
To compare differences in peak severity of illness.	The scoring system is described in section 9.6.1 reflects the severity of respiratory illness. The maximum severity score during the entire study period will be compared.	Ascertain from day 14 and day 28 telephone call and from retrospective ePR/medical notes data at 28 days after randomisation.
Safety and tolerability	Serious adverse events and concomitant medications. Record at enrolment, emergently during study period and proactively elicit at day 14 and at day 28.	Emergent data collection days 0-28 and elicit proactively at day 14 and day 28 post randomisation.
**Exploratory Objectives**<br>Mechanistic analysis of blood and nasal biomarkers if available	The following samples may be taken. Blood for serum, Tempus tube (whole blood RNA), EDTA tubes (PBMC), nasal brush to be placed immediately into RNA lysis buffer (for subsequent PCR and transcriptomic analysis).	Samples to be collected prospectively at baseline and again if patient admitted, to be taken as soon as possible and within 72 hours of admission if possible.

© Copyright: The University of Oxford and Oxford University Hospitals NHS Foundation Trust 2019

7 TRIAL DESIGN

Study design: Multi centre, prospective open label two-arm randomised superiority clinical trial of standard care and Azithromycin with standard care alone for those presenting to hospital with COVID-19 symptoms who are not admitted at initial presentation.

Study setting: Patients being assessed by secondary care NHS hospitals in the UK.

Participants: Adults, ≥18 years of age assessed in an acute hospital with clinical diagnosis of COVID-19 infection and where medically it is decided not to admit the patient and for the patient to be managed on an ambulatory (outpatient) care pathway at their usual residence (home or care home).

Study schedule: Enrolment on day 0. Telephone follow up at day 14 day, and day 28. If admitted between randomisation and day 28, data will be collected until hospital discharge (Figure 2, Appendix B).

Intervention: Azithromycin 500 mg orally once daily for 14 days. The first dose will be within 4 hours of randomisation. This is in addition to standard care as per local hospital advice for those patients with suspected COVID who are not admitted: i.e. symptomatic relief with rest, as-required paracetamol (where appropriate) and advice to seek further medical attention if significant worsening of breathlessness.

Comparator: Standard care as per local hospital advice for those patients with suspected COVID who are not admitted: i.e. symptomatic relief with rest, as-required paracetamol (where appropriate) and advice to seek further medical attention if significant worsening of breathlessness.

Data collection: Study data will be collected as per the study schedule. Serious adverse events and concomitant medications will be monitored from Day 0 until Day 28 via the day 14 and day 28 contacts made to participants, any direct contacts to the central trial team via the study contact details or via patient/notes reviews at sites if participants are admitted. Severity Scale for Clinical Improvement scores (see section 9.6.2) will be collected at days 0, 14 and 28 for patients who remain in an ambulatory pathway and daily for patients who are admitted until hospital discharge and at day 14 and day 28. Data will be collected by face-to-face discussion and assessment of patient (for hospital admissions), or by telephone discussion (for those ambulatory patients), as well as from retrospective patient records/notes and radiology results (for all patients).

© Copyright: The University of Oxford and Oxford University Hospitals NHS Foundation Trust 2019

Study Schedule:

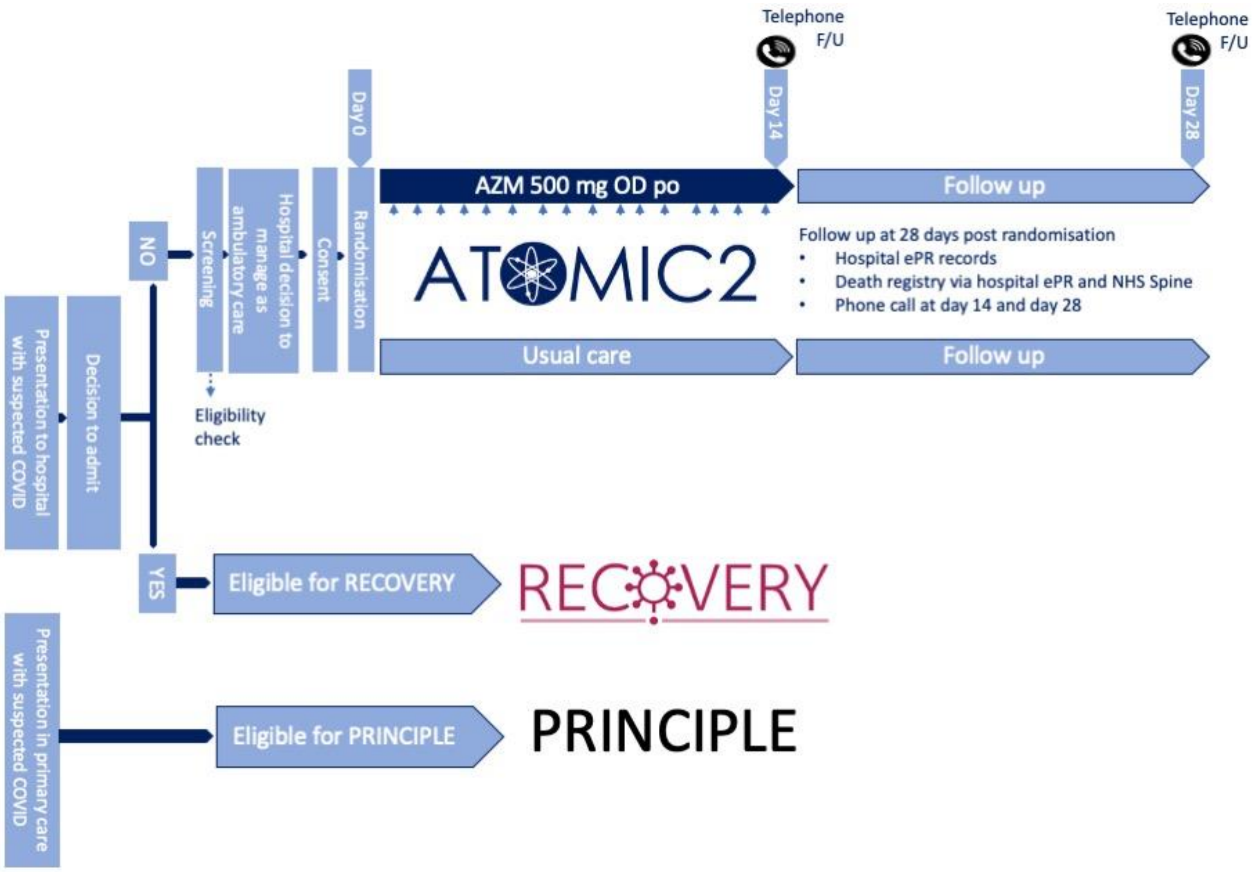

Note – there is a national trial open in the UK recruiting called RECOVERY this only recruits patients who are hospitalised with COVID-19 – therefore ATOMIC2 is not competing for patients.

Sample size:

The definitive trial will recruit approximately 800 participants (400 per arm). An interim analysis has been built into this trial after an initial 100 patients have been randomised, treated and followed-up for 28 days. The DSMC will review the accruing safety and efficacy data and the results from a futility analysis, which will assess whether the trial would be likely to confirm superiority of the active treatment if it was to continue as planned. They will provide recommendations to the TSC as to whether the trial should continue to the definitive trial or stop early for safety or futility.

Note: Whilst the interim analysis is prepared for and undertaken, and the DSMC meeting held – recruitment will continue to the trial as per protocol.

If the recommendation is to continue to the definitive trial they will also review the assumption on which the sample size is based, blinded to treatment allocation, and the final sample size for the definitive study will be confirmed by the DSMC and TSC. This is a rapidly evolving disease area

© Copyright: The University of Oxford and Oxford University Hospitals NHS Foundation Trust 2019

and information about the control rate for progression to hospitalisation or death is not yet fully known. More details will be available during the trial and will be assessed to refine the sample size. Initial assumptions are described below.

The total sample size of 778 participants with primary outcome data are required to reject the null hypothesis of no difference between the active treatment and usual care. This number is based on the following assumptions: 30-40% of patients following usual care will progress to hospitalisation or death within 28 days; 90% power, 2-sided 5% significance and a 30-35% reduction in progression or death for patients on Azithromycin (778 participants required to detect a 33.3% reduction from 30% to 20% in progression to hospitalisation or death or 646 required to detect a 30% reduction from 40% to 28% in progression to hospitalisation or death). To allow for uncertainty around the assumptions and allowing for a 2% loss to follow-up, approximately 800 participants will be required. Total sample size for the definitive study will be refined at the interim analysis if progression to the full trial is the option chosen.

Study duration:
Treatment duration 14 days.
Duration of follow up 28 days from randomisation, unless admitted to hospital within 28 days of randomisation, then the participant will be followed until their discharge from hospital.
Duration of study: Anticipated recruitment period up to 6 months

8 PARTICIPANT IDENTIFICATION

8.1 Trial Participants

Adults, ≥18 years of age assessed in an acute hospital with clinical diagnosis of COVID-19 infection.

8.2 Inclusion Criteria

Patients are eligible for the study if all of the following are true:

- Male or Female, aged at least 18 years
- Assessed by the attending clinical team as appropriate for initial ambulatory (outpatient) management
- A clinical diagnosis of highly-probable COVID-19 infection (diagnosis by the attending clinical team)
- No medical history that might, in the opinion of the attending clinician, put the patient at significant risk if he/she were to participate in the trial
- Able to understand written English (for the information and consent process) and be able to give informed consent

© Copyright: The University of Oxford and Oxford University Hospitals NHS Foundation Trust 2019

8.3 Exclusion Criteria

The participant may not enter the trial if ANY of the following apply:

- Known hypersensitivity to any Macrolide including Azithromycin, Ketolide antibiotic, or the excipients including an allergy to soya or peanuts.
- Known fructose intolerance, glucose-galactose malabsorption or sucrose-isomaltase-insufficiency
- Currently on a Macrolide antibiotic (Clarithromycin, Azithromycin, Erythromycin, Telithromycin, Spiramycin)
- On any SSRI (Selective Serotonin Reuptake Inhibitor)
- Elevated cardiac troponin at initial assessment suggestive of significant myocarditis (if clinically the clinical team have felt it appropriate to check the patient's troponin levels)
- Evidence of QTc prolongation: QTc>480ms
- Significant electrolyte disturbance (e.g. hypokalaemia K+<3.5 mmol/L)
- Clinically relevant bradycardia (P<50 bpm), non-sustained ventricular tachycardia or unstable severe cardiac insufficiency
- Currently on hydroxychloroquine or chloroquine

9 TRIAL PROCEDURES

For schedule of procedures see Appendix B.

9.1 Recruitment

Patients will be identified by the clinical care teams as clinically diagnosed highly-probable COVID-19 in acute ambulatory care units, acute medical units and emergency departments within acute hospitals. A member of clinical care team will then screen the patient for eligibility and discuss the trial with the patient, or alternatively, they will ask the patients if a member of the research team could approach them to talk to them about ATOMIC2.

9.2 Screening and Eligibility Assessment

Any patient randomised must be able to receive the first dose of Azithromycin within 4 hours of randomisation. Protocol waivers are not permitted.

Patients will be screened by review of ePR/medical notes data for history of presentation, examination findings, ECG result, K^+ and, concomitant medications by anyone listed on the site study delegation log.

© Copyright: The University of Oxford and Oxford University Hospitals NHS Foundation Trust 2019

9.3 Informed Consent

Informed consent will be obtained from each patient before enrolment into the study, by a member of the study team who is listed on the delegation log for taking informed consent.

Written and verbal versions of the Participant Information and Informed Consent will be presented to the participants detailing no less than: the exact nature of the trial; what it will involve for the participant; the implications and constraints of the protocol; the known side effects and any risks involved in taking part. It will be clearly stated that the participant is free to withdraw from the trial at any time for any reason without prejudice to future care, without affecting their legal rights and with no obligation to give the reason for withdrawal.

The participant will be allowed time to consider the information, and the opportunity to question the Investigator, however due to the nature of the disease and limited time available, they may not be time for the individual to contact their GP or other independent parties to decide whether they will participate in the trial, as individuals can only enter the study whilst being reviewed in the COVID-19 areas of hospital, individuals cannot go home and then decide to take part in the study.

Written Informed Consent will then be obtained by means of participant dated signature and dated signature of the person who presented and obtained the Informed Consent. The person who obtained the consent must be suitably qualified and experienced, and have been authorised to do so by the Chief/Principal Investigator. Consent will be taken electronically, and a copy of the patient's consent form will be emailed to the study site and the patient from a secure NHS email address. Consent will be requested after presentation of the trial Patient Information Sheet and a discussion has been had with the patient.

9.4 Randomisation

A medically qualified doctor must confirm that a patient is eligible for this CTIMP. Eligible patients will be randomised using the centralised validated computer randomisation program through a secure (encrypted) web-based service, RRAMP (https://rramp.octru.ox.ac.uk), provided by the Oxford Clinical Trials Research Unit (OCTRU), accessed via the study's RedCap instance, with a minimisation algorithm to ensure balanced allocation across treatment groups, stratified by centre, hypertension (yes/no), diabetes (yes/no) and sex (male/female) in a 1:1 ratio to either Azithromycin or usual care. To ensure the unpredictability of treatment allocation the minimisation algorithm will include a probabilistic element and a small number of participants randomised by simple randomisation.

Note: Hypertension is defined as any hypertension previously diagnosed by a doctor prior to presenting to the hospital with COVID-19 symptoms.
Note: Diabetes is defined as any diabetes that is treated with oral or injectable therapy.

© Copyright: The University of Oxford and Oxford University Hospitals NHS Foundation Trust 2019

Stratification by centre will help to ensure that any centre-effect will be equally distributed in the trial arms and enable practical issues associated with the active intervention to be overcome.

There is some emerging evidence that patients who have underlying hypertension, diabetes and are male are more likely to progress and require hospitalisation, so it is important for the two treatments to be balanced across these potentially important prognostic factors.

The following information will be recorded on a secure web-based form in the study randomisation system (RRAMP) by the attending clinician or delegate including a member of research team to enable follow-up:

- Patient details e.g. name, NHS number, date of birth, sex, telephone number and GP details

Note: These data fields will allow sites to check their local hospital records and/or NHS Spine (or devolved nation equivalents) to check that a patient has not died or been admitted to avoid any upset of patient's relatives. The GP details are required to allow the central trial team to send a letter to the patient's GP informing them of their ATOMIC2 participation.

- *An email address will also be recorded to enable a copy of the completed consent form to be sent to the patient or at their request a different individual for safe keeping.*

9.5 Blinding and code-breaking

This is not applicable, as this is an open label study without blinding.

9.6 Baseline Assessments (Day 0)

The following information will be recorded on the web-based form which goes straight into the password protected study database by the attending clinician or delegate including a member of research team:

- COVID-19 symptom onset date
- Presence or absence of COVID -19 symptoms using COVID COS scales (0=no, 1=mild, 2=moderate, 3=significant)
  - Shortness of Breath
  - Fever (Temperature ≥ 37.8°C, oral/rectal or tympanic)
  - Loss of taste
  - New persistent cough
  - Diarrhoea
  - Body pain
  - Changes to sense of smell
  - Fatigue
- COVID-19 symptoms history
- Latest vital signs (HR, RR, Pulse Oximetry, BP, Temperature)
- Major comorbidity and medical history
  - cardiovascular disease
  - diabetes,
  - chronic lung disease
  - asthma
  - hypertension
  - ongoing cancer treatment

© Copyright: The University of Oxford and Oxford University Hospitals NHS Foundation Trust 2019

- Date of initial assessment in an acute hospital
- Smoking status
- Ethnicity
- Occupation (including specifically whether a HealthCare or Laboratory worker)
- Charlson Index
- Severity score (as per section 9.6.1)
- Concomitant medications (specifically prednisolone, inhaled corticosteroids, ACE inhibitors, antibiotics)
- Results of chest auscultation
- CXR results i.e presence or absence of pneumonia
- Usual residence (home or residential care)
- Number of members in household
- Number of members including participants currently showing any COVID-19 symptoms
- If an oropharyngeal viral swab for COVID-19 was taken
- Usual care biochemistry results from hospital (U+E, FBC, Fibrinogen, D-Dimer, APTT, PT, Troponin)
- 12 lead ECG as part of clinical care
- If the participant is known to be pregnant
- If the participant is lactating

9.6.1 Severity scale score for peak severity of illness

The Severity scale score should be given based on clinical condition. If a patient is hospitalised for reasons of isolation or quarantine they should not automatically be given the score of 3, ascribe the score which is relevant to their clinical status, which maybe for example ambulatory score 1 even if they are hospitalised. The highest score obtained during the 28 day study period will be used in the final analysis, based on data obtained at 14 and 28 days.

**Descriptor**	**Score**
Ambulatory. No limitation of activities	0
Limitation of simple activities	1
Hospitalised, mild disease, no oxygen therapy	2
Hospitalised, oxygen by conventional delivery system[1] ≤40% mask or nasal prongs	3
Hospitalised, oxygen by conventional delivery system[1] >40% mask	4
Hospitalised receiving non-invasive ventilation or receiving high-flow oxygen therapy (HFOT, >15 L/min), or continuous positive airway pressure (CPAP)[2]	5
Intubation and mechanical ventilation[3]	6
Ventilation + additional organ support	7
Death	8

[1] Criteria filled if oxygen is required to maintain saturations >92% or above normal baseline for patients who use home oxygen [2] Any patient requiring any form of PEEP delivery, or those in whom such devices are not tolerated requiring >50% oxygen with a RR>25, or rising CO_2 in the absence of known lung disease

© Copyright: The University of Oxford and Oxford University Hospitals NHS Foundation Trust 2019

[3] In patients unsuitable for ventilation, criterion is met when requiring >50% oxygen with a RR>25, or rising CO_2.

9.7 Subsequent assessments

Subsequent assessments will be carried out at days 14 and 28 by telephone call.

The following information will be recorded:

- For those randomised to AZM - date of starting treatment
- Presence of COVID -19 symptoms using COVID COS scales 0=no, 1=mild, 2=moderate, 3=significant)
    - Shortness of Breath
    - Fever (Temperature ≥ 37.8°C, oral/rectal or tympanic)
    - Loss of taste
    - New persistent cough
    - Diarrhoea
    - Body pain
    - Changes to sense of smell
    - Fatigue
- (At 14 day call only) For those randomised to AZM – number of tablets remaining
- COVID-19 symptoms history for past 14 days
- COVID-19 swab results from hospital records if a swab was taken
- Any change to concomitant medications
- Worse severity scale score in previous 14 days
- Any visits to Hospital due to COVID-19 symptoms in previous 14 days
- Any adverse events

There will be a mortality check at day 14 and day 28 **before any contact is made with participants**, using hospital systems and NHS Spine or equivalent devolved nation systems – it is anticipated that because the UK Government COVID-19 lockdown regulations prohibit travel and in the vast majority of cases patients will be readmitted to their local hospital after deterioration. If on calling the participant at day 14 or day 28 the participant has been readmitted, data will be collected by hospital note review instead of from the participant. The following data will be extracted:

- Date of admission
- Name of hospital
- Any level 2 ventilation received – duration, type, date initiated
    - Reason for stopping level 2 ventilation – Need for level 3 IMV, patient improvement, died
- Any level 3 ventilation received – duration, type, date initiated
    - Reason for stopping level 3 ventilation – Died, patient improvement, requirement by others for equipment
- Pneumonia – diagnosis, severity (using BTS CURB-65 score).

© Copyright: The University of Oxford and Oxford University Hospitals NHS Foundation Trust 2019

- Daily severity scale score
- Date of discharge from hospital, location of discharge
- Any adverse events
- Concomitant medications (specifically prednisolone, inhaled corticosteroids, ACE inhibitors, antibiotics, antivirals and antifungals)
- Any complications from COVID-19 occurring during admission
- Any other COVID-19 trials participated in

If participating sites allow, have staff available with suitable experience, equipment and time, then the following samples are optional to be given by participants, for the study's exploratory outcomes.

9.7.1 At study recruitment (optional for all sites and participants)

- EDTA tube to be frozen for subsequent DNA analysis. (4ml blood)
- Serum SST tube to be frozen (5ml blood)
- Tempus blood RNA tube to be frozen (3ml blood)
- Nasal sampling using a small brush into lysis buffer to be frozen
- EDTA tubes for cell preparation of peripheral blood mononuclear cells to be frozen (1 x 10ml EDTA tubes, 1 x 4ml EDTA tube)

On hospital admission if it occurs within 28 days of randomisation the following samples will be taken (optional for all sites and participants)

- Serum SST tube to be frozen (5ml blood)
- Tempus blood RNA tube to be frozen (3ml blood)
- Nasal sampling using a small brush into lysis buffer to be frozen
- EDTA tubes for cell preparation of peripheral blood mononuclear cells to be frozen (1 x 10ml EDTA tubes, 1 x 4ml EDTA tube)

9.7.2 Sample handling, processing and analysis

A sample handling manual will be provided separately. The trial team will have access to the samples. Samples may be used for analysis of immunological, virological and genetic parameters. If whole genome sequencing is performed to determine predisposition to severe disease, incidental findings will be of unknown significance to participants and will not be made available to them unless they are directly related to a relevant immune defect or of relevance to COVID-19. De-identified samples might be analysed by commercial organisations.

De-identified samples, where consent is in place, may be used for future ethically approved studies.

Note: If a COVID-19 swab is taken at site that is not tested locally – these swabs will be stored in Category 3 freezers at site, then transferred to the University of Oxford at the end of the study in

© Copyright: The University of Oxford and Oxford University Hospitals NHS Foundation Trust 2019

a controlled manner. If the COVID-19 swab is processed at the hospital, the results of this test will be extracted from the patients' medical notes/ePR.

9.8 GP notification

Participants will be asked for permission for their GP to contacted to notify them of their participation in the study. As this study is open label, GPs will be informed of whether the participant has been dispensed a 14 day course of Azithromycin or no drug. No further communications will be made with the GP as any hospital admission and treatment will result in standard hospital discharge paperwork being automatically sent to the GP.

9.9 Early Discontinuation/Withdrawal of Participants

During the course of the trial a participant may choose to withdraw early from the trial treatment at any time. This may happen for a number of reasons, including but not limited to:

- Inability to comply with trial procedures
- Participant decision

Participants may choose to stop treatment but may remain on study follow-up.

Each participant has the right to withdraw their consent to continue from the study at any time. The reason for withdrawal will be asked and this will be recorded in the CRF, but the participant is not obliged to give a reason. In the event of a discontinuation from the trial during the 14 days following randomisation, participants will be asked to stop taking the IMP. Participants may withdraw from active follow-up and further communication but allow the trial team to continue to access their medical records and any relevant hospital data that is recorded as part of routine standard of care; i.e., CT-Scans, blood results and disease progression data etc. Participants may request that samples they have donated be destroyed.

In addition, the Investigator may discontinue a participant from the trial treatment at any time if the Investigator considers it necessary for any reason including, but not limited to:

- Development of new significant hepatic dysfunction (See Cautions, section 10.1.5)
- Development of severe cardiac dysfunction.
- Ineligibility (either arising during the trial or retrospectively having been overlooked at screening)
- Significant non-compliance with treatment regimen or trial requirements

If the participant is withdrawn due to a serious adverse event, the Investigator will arrange for follow-up until the adverse event has resolved or stabilised.

© Copyright: The University of Oxford and Oxford University Hospitals NHS Foundation Trust 2019

9.10 Definition of End of Trial

The end of trial will be when all samples taken have been analysed and all the data has been entered into the clinical database and all queries have been resolved.

10 SAFETY REPORTING

10.1 Adverse Event Definitions

Adverse Event (AE)	Any untoward medical occurrence in a participant to whom a medicinal product has been administered, including occurrences which are not necessarily caused by or related to that product.
Adverse Reaction (AR)	An untoward and unintended response in a participant to an investigational medicinal product which is related to any dose administered to that participant.<br>The phrase "response to an investigational medicinal product" means that a causal relationship between a trial medication and an AE is at least a reasonable possibility, i.e. the relationship cannot be ruled out.<br>All cases judged by either the reporting medically qualified professional or the Sponsor as having a reasonable suspected causal relationship to the trial medication qualify as adverse reactions.
Serious Adverse Event (SAE)	A serious adverse event is any untoward medical occurrence that:<br>• results in death<br>• is life-threatening<br>• requires inpatient hospitalisation or prolongation of existing hospitalisation<br>• results in persistent or significant disability/incapacity<br>• consists of a congenital anomaly or birth defect*.<br>Other 'important medical events' may also be considered a serious adverse event when, based upon appropriate medical judgement, the event may jeopardise the participant and may require medical or surgical intervention to prevent one of the outcomes listed above.

© Copyright: The University of Oxford and Oxford University Hospitals NHS Foundation Trust 2019

	NOTE: The term "life-threatening" in the definition of "serious" refers to an event in which the participant was at risk of death at the time of the event; it does not refer to an event which hypothetically might have caused death if it were more severe.
Serious Adverse Reaction (SAR)	An adverse event that is both serious and, in the opinion of the reporting Investigator, believed with reasonable probability to be due to one of the trial treatments, based on the information provided.
Suspected Unexpected Serious Adverse Reaction (SUSAR)	A serious adverse reaction, the nature and severity of which is not consistent with information in the MHRA approved Reference Safety Information

Note: To avoid confusion or misunderstanding of the difference between the terms "serious" and "severe", the following note of clarification is provided:

"Severe" is often used to describe intensity of a specific event, which may be of relatively minor medical significance.

"Seriousness" is the regulatory definition supplied above.

Records of adverse events and concomitant medications, recorded at day 14 (till 24 h after last dose) and at day 28. In the event of an adverse event the Investigator will arrange for telephone calls until the adverse event has resolved or stabilised.

10.2 Reportable Events for ATOMIC2

As discussed in section 10.4.1 only those events which are serious and related to AZM treatment are defined as reportable events for this trial for the purpose of regulatory safety reporting. Disease progression is reported as a trial outcome.

10.3 Assessment of Causality

The relationship of each reportable adverse event to the trial medication must be determined by a medically qualified individual according to the following definitions:

Unrelated – Where an event is not considered to be related to the IMP / intervention

Possibly Related – although a relationship to the IMP / intervention cannot be completely ruled out, the nature of the event, the underlying disease, concomitant medication or temporal relationship make other explanations possible.

© Copyright: The University of Oxford and Oxford University Hospitals NHS Foundation Trust 2019

Probably Related – the temporal relationship and absence of a more likely explanation suggest the event could be related to the IMP / intervention

Definitely Related – the known effects of the IMP, its therapeutic class or based on challenge testing suggests that the IMP / intervention is the most likely cause.

10.4 Procedures for Reporting Adverse Events

Azithromycin is a very well known, commonly used drug with a well-known safety profile. The aim of this trial is to test the effectiveness of the drug is preventing deterioration, not explicitly assessing safety of this well used drug. AEs considered related to the trial medication as judged by a medically qualified investigator or the Sponsor will be followed either until resolution, or the event is considered stable. The following information will be recorded: description, date of onset and end date, severity, assessment of relatedness to trial medication and action taken. Follow-up information should be provided as necessary. The severity of events will be assessed on the following scale: 1 = mild, 2 = moderate, 3 = severe.

It will be left to the Investigator's clinical judgment to decide whether or not an AE is of sufficient severity to require the participant's removal from treatment. A participant may also voluntarily withdraw from treatment due to what he or she perceives as an intolerable AE. If either of these occurs, the participant will be followed-up and be given appropriate care under medical supervision until symptoms cease, or the condition becomes stable.

10.5 Reporting Procedures for Serious Adverse Events

All SAEs (other than those defined as foreseeable in section 10.5.1 below) occurring within the first 14 days of the IMP administration will be reported. All SAE information must be recorded on an ATOMIC2-Trial specific SAE form and scanned and emailed to OCTRU within 24 hours of the Site Study Team becoming aware of the event. OCTRU will perform an initial check of the report, request any additional information and forward to a Medical Reviewer (Nominated Person as per OCTRU SOP) for review.

Additional and further requested information (follow-up or corrections to the original case) will be detailed on a new SAE Report Form and emailed to OCTRU.

10.5.1 Events exempt from reporting as SAEs

It is important to consider the natural history of COVID-19 with regards to this study, the expected sequelae of the illness, and the relevance of these complications to the trial treatment. All eligible participants have a potential poor prognosis, and due to the complexity of their condition are at increased risk of experiencing multiple adverse events. Additionally, Azithromycin has a very well known safety profile. Therefore taking a risk adapted response, the labelling of a Serious Adverse Event (SAE) will be limited to serious events, which might

© Copyright: The University of Oxford and Oxford University Hospitals NHS Foundation Trust 2019

reasonably occur as a consequence of the trial treatment (i.e. not events that are part of the natural history of the primary disease process, such as hospitalisation or death). SAEs must be reported in the participant's medical notes, on the trial CRF, and reported to the CTU using the SAE Reporting Form, within 24 hours of the site observing or learning of the SAE(s). All sections of the SAE Reporting Form must be completed.

Deaths due to COVID-19 disease under study are exempt from reporting as SAEs as they will be captured as part of the primary outcome. An AE should not be recorded for the positive SAR-CoV-19 infection, this will be known at time of inclusion into the study and should be recorded as medical history. Worsening of COVID-19 symptoms is captured as an efficacy measure and in general will not be considered an adverse event.

10.5.2 Procedure for immediate reporting of Serious Adverse Events

- Site study team will complete an SAE report form for all reportable SAEs.
- Where the SAE requires immediate reporting, the SAE report form will be scanned and emailed to The Trial Manager or delegate immediately i.e., within 24 hours of site study team becoming aware of the event using atomic2@ndorms.ox.ac.uk or using the study CRF system.
- Site study team will provide additional, missing or follow up information in a timely fashion.

10.6 Expectedness

Assessment of Expectedness will be determined centrally by the Nominated Person and according to the current MHRA approved RSI section of the Summary of Product Characteristics.

10.7 SUSAR Reporting

All SUSARs will be reported by the sponsor delegate to the relevant Competent Authority and to the REC and other parties as applicable. For fatal and life-threatening SUSARS, this will be done no later than 7 calendar days after the Sponsor or delegate is first aware of the reaction. Any additional relevant information will be reported within 8 calendar days of the initial report. All other SUSARs will be reported within 15 calendar days.

Principal Investigators will be informed of any SUSARs with this IMP at the same time as reporting to the MHRA and REC.

10.8 Development Safety Update Reports

The CI will submit (in addition to the expedited reporting above) DSURs once a year throughout the clinical trial, or on request, to the Competent Authority (MHRA in the UK), Ethics Committee, HRA (where required) and Sponsor.

© Copyright: The University of Oxford and Oxford University Hospitals NHS Foundation Trust 2019

11 TRIAL INTERVENTIONS

11.1 Investigational Medicinal Product(s) (IMP) Description

11.1.1 Study intervention

Subjects will be randomised to receive Azithromycin 500 mg daily orally for 14 days or standard care. The first dose will be taken within 4 hours of randomisation. Participants will be asked to take the AZM at the approximately the same time every day for 14 days. The drug should be taken ideally 1 hour before a meal or 2 hours afterwards. Participants will be asked to take 250mg capsules each day to ensure a dose of 500mg is taken.

The comparator will be usual care. i.e. symptomatic relief with rest, as-required paracetamol (where appropriate) and advice to seek further medical attention if significant worsening breathlessness. No specific therapies are yet available for COVID-19. Should additional interventions become evidence-based standard practice during the conduct of this study these would also be permitted to be provided and will be recorded in the concomitant medications.

11.1.2 Dose regimen

Due to its long half-life AZM accumulates over time, but to achieve a rapid effect we will use 500mg OD for 14 days, similar to the dose recommended for Lyme disease[44].

11.1.3 Authorisation and safety

Azithromycin has a marketing authorisation in the UK and in EU member states. Azithromycin is generally well-tolerated with a very good and well-documented safety record. Even in long term administration (500mg thrice weekly for 48 weeks, n=213 individuals, median age 61y) there was no increase in serious adverse events v placebo, the main adverse event being an increase in diarrhoea (34% v 19% not associated with study withdrawal)[7]. The main adverse event of concern would be potential cardiovascular toxicity. Although macrolides have a class warning for potential cardiac QT prolongation, Azithromycin does not show this effect under experimental conditions[45]. Only a few cases of QT prolongation have been reported for patients treated with the drug[46], mainly because Azithromycin, unlike other macrolide antibiotics, does not interact with CYP3A4, despite a minor interaction with the anti-coagulant warfarin[47]. In the large AMAZES RCT there was no increase in QTc prolongation, although this study excluded participants with QTc>480ms[7]. Recently a large study of Medicaid prescriptions reported an additional risk of cardiovascular death of 47 extra deaths / million v amoxicillin (relative risk (RR) for cardiovascular death 2.49[48], and a meta-analysis of 20 million patients suggested a RR for cardiac death or VT of 2.42[49]. However these effects are very small and subject to confounding, and at odds with more recent studies: in a review of 185,000 Medicare patients odds ratio for CV death was only 1.35, and after controlling for covariates decreased to 1.01 (0.95-1.08)[50], whilst a large Cochrane review of 183 trials found no evidence of an increase in cardiac disorders with macrolides (OR 0.87)[51]. Overall

© Copyright: The University of Oxford and Oxford University Hospitals NHS Foundation Trust 2019

the risk to a patient treated would be low compared with the considerable mortality of COVID-19, particularly if patients with QTc>480ms were excluded.

11.1.4 Contraindications

Hypersensitivity to the active substance, any macrolide, ketolide antibiotic, or the excipients. Known fructose intolerance, glucose-galactose malabsorption or sucrose-isomaltase-insufficiency.

11.1.5 Cautions

Use with caution in patients with ongoing proarrhythmic conditions (Prolonged QT, coadministration with quinidine, procainamide, dofetilide, amiodarone, sotalol, cisapride, terfenadine, antipsychotic agents such as pimozide; antidepressants such as citalopram; and fluoroquinolones such as moxifloxacin and levofloxacin).

Caution with electrolyte disturbance, particularly in cases of hypokalaemia; with clinically relevant bradycardia, non-sustained ventricular tachycardia or unstable severe cardiac insufficiency.

Caution with hepatic dysfunction. Hepatic dysfunction is common in COVID-19 disease, with elevation of alanine aminotransferase (ALT) or aspartate aminotransferase (AST) occurring at some stage during disease in 14–53% of hospitalised cases[52]. However this is typically mild and clinically significant liver injury is uncommon even if severe patients[53]. There is no evidence later presentation is associated with worse hepatic derangement[53]. Mild hepatic dysfunction is not a contraindication to AZM prescription. The SmPC by Pfizer states: A dose adjustment is not necessary for patients with mild to moderately impaired liver function. Since liver is the principal route of elimination for Azithromycin, the use of Azithromycin should be undertaken with caution in patients with significant hepatic disease. Cases of fulminant hepatitis potentially leading to life-threatening liver failure have been reported with Azithromycin. Some patients may have had pre-existing hepatic disease or may have been taking other hepatotoxic medicinal products. In case of signs and symptoms of liver dysfunction, such as rapid developing asthenia associated with jaundice, dark urine, bleeding tendency or hepatic encephalopathy, liver function tests / investigations should be performed immediately[54]. Azithromycin administration should be stopped if significant liver dysfunction (e.g. AST and/or ALT >5x upper limit of normal[55]) has emerged since commencing the drug.

Coumarin-type oral anticoagulants: The SmPC states in a pharmacokinetic interaction study, azithromycin did not alter the anticoagulant effect of a single dose of 15 mg warfarin administered to healthy volunteers. There have been reports received in the post-marketing period of potentiated anticoagulation subsequent to co-administration of azithromycin and coumarin-type oral anticoagulants. Although a causal relationship has not been established, consideration should be given to the frequency of monitoring prothrombin time when azithromycin is used in patients receiving coumarin-type oral anticoagulants. It should also be noted that many potential

© Copyright: The University of Oxford and Oxford University Hospitals NHS Foundation Trust 2019

participants may be prescribed other antibiotics as part of standard care. Therefore concomitant use of coumarins is not contraindicated, but it is recommended in the PIS that if prescribed azithromycin or any other antibiotic participants should attend their GP surgery for a repeat INR check after 3 to 5 days to check.

Nelfinavir: The SmPC states: Co-administration of azithromycin (1200 mg) and nelfinavir at steady state (750 mg three times daily) resulted in increased azithromycin concentrations. No clinically significant adverse effects were observed and no dose adjustment was required. Concomitant use of nelfinavir is therefore not contraindicated.

Trimethoprim/sulfamethoxazole: Co-administration of trimethoprim/sulfamethoxazole DS (160 mg/800 mg) for 7 days with 1200mg azithromycin on Day 7 had no significant effect on peak concentrations, total exposure or urinary excretion of either trimethoprim or sulfamethoxazole. Azithromycin serum concentrations were similar to those seen in other studies. Therefore concomitant use of co-trimoxazole is not contraindicated.

11.1.6 Pregnancy

The SmPC states only to be used during pregnancy if the benefit outweighs the risk. Given the high (30-40%) mortality of this condition in hospitalised patients, and lack of teratogenic effect of azithromycin in animal studies, large human database studies including >1000 live births[56], or post-marketing surveillance[57], pregnancy will not be a contraindication to the study medication, but this decision will be subject to the principal investigator's discretion. Lactation: avoid breastfeeding till 2 days after discontinuation of treatment.[54]

Pregnancy itself is not an AE unless there is a suspicion that the study medication may have interfered with the effectiveness of a contraceptive medication.

11.1.7 Blinding of IMPs

This is an open-label study. However, while the study is in progress, access to tabular results by allocated treatment allocation will not be available to the research team, patients, or members of the Steering Committee (unless the DSMC advises otherwise).

11.1.8 Storage and dispensing of IMP

Commercial stock of study medication will be delivered to participating hospital pharmacies. Depending on supplies, the drug is either provided in blister packs or 100 capsules/bottle.

Hospital pharmacies will work under Exemption 37 to assemble the drug for individual use and will add the approved ATOMIC2 clinical trial label. A copy of the standard drug patient information leaflet normally supplied with Azithromycin will be also be provided.

Once Pharmacy prepares the labelled drug packs these will be sent for secure storage to the acute medicine areas where patients are being triaged. The prescribing doctor will complete the details

© Copyright: The University of Oxford and Oxford University Hospitals NHS Foundation Trust 2019

of the patient and of the dispense date on the label. The research team will also complete drug accountability logs.

11.1.9 Compliance with Trial Treatment

Compliance will be assessed by telephone discussion with patient on day 14 of treatment with specific questioning as to the number of pills remaining. Adequate compliance will be defined as the first dose being administered within 4 hours of randomisation and at least 80% of doses i.e. a maximum of 4 / 28 tablets remaining at the end of day 14.

11.1.10 Concomitant Medication

Cautions and precautions summarised in the SmPC will be followed during this trial. Co-administration of the following medications will not be allowed and will be criteria for exclusion or withdrawal from the trial treatment: quinidine, procainamide, dofetilide, amiodarone, sotalol, cisapride, terfenadine, antipsychotic agents such as pimozide; antidepressants such as citalopram; fluoroquinolones such as moxifloxacin, levofloxacin and ciprofloxacin; chloroquine and hydroxychloroquine; use of another macrolide antibiotic (clarithromycin, erythromycin, azithromycin, telithromycin, spiramycin).

11.1.11 Post-trial treatment

There will be no provision of the IMP beyond the trial period.

11.2 Other Treatments (non-IMPs)

None.

11.3 Other Interventions

There are no other interventions in the trial design.

To assess safety and tolerability: records of adverse events and concomitant medications, reported emergently to the study team and directly elicited on days 14 and 28 at telephone follow-up. In the event of an adverse event the Investigator will arrange for follow-up visits or telephone calls until the adverse event has resolved or stabilised.

12 STATISTICS

12.1 Statistical Analysis Plan (SAP)

Full details of the statistical analysis will be detailed in a separate statistical analysis plan (SAP) which will be drafted early in the trial and finalised prior to the interim analysis data lock. Stata (StataCorp LP) or other appropriate validated statistical software will be used for analysis. A summary of the planned statistical analysis is included here.

© Copyright: The University of Oxford and Oxford University Hospitals NHS Foundation Trust 2019

12.2 Description of Statistical Methods

Standard descriptive statistics will be used to describe the demographics between the treatment groups reporting means and standard deviations or medians and interquartile ranges as appropriate for continuous variables and numbers and percentages for binary and categorical variables.

The proportion of patients progressing to hospitalisation or death by day 28 post-randomisation is the primary outcome for this study. The difference in proportions between the treatment arms will be assessed using a chi-squared tests and a 5% (2-sided) significance level. Difference in proportions together with the 95% confidence intervals will be reported. Adjusted analyses will also be undertaken using logistic regression with progression as the binary outcome, adjusting for stratification factors (centre, hypertension, diabetes and sex) and other important prognostic variables, which will be fully defined in the SAP. Time to event analysis will also be undertaken to explore whether the active treatment delays progression. The success (or otherwise) of the trial will be based on the adjusted analysis. Both relative and absolute differences in proportions will be reported together with 95% confidence intervals.

Other binary outcomes will be assessed using similar methods and continuous variables will be assessed using linear regression analysis. Where appropriate longitudinal methods will incorporate different time points.

The number and percentage of subjects with each score on the severity scale for clinical improvement will be presented at baseline and each post baseline time point. In addition, the change in severity scale score from baseline will be summarised on both a categorical scale, using counts and percentages and on a continuous scale using descriptive statistics. Inferential statistical analyses such as ordered logistic regression, mixed model for repeated measurement (MMRM) or Mann-Whitney may be conducted in an exploratory fashion to aid the understanding of the data. Binary interpretations of the severity scale for clinical improvement may also be defined in the SAP, such as responders (any improvement at day 14) and complete responders (score of 0 at day 14) and these will be compared using logistic regression as for the primary outcome.

12.3 Sample Size Determination

This trial will recruit up to 800 participants (400 per treatment arm) with an interim analysis built in to review safety and efficacy data after an initial 100 patients have been randomised, treated and followed-up for 28 days. Following this interim review the trial maybe stopped for safety or futility (i.e. the trial is unlikely to find that Azithromycin is superior). If not stopped early, the assumptions on which the sample size is based will be reviewed blinded to the treatment allocation and the final sample size for the definitive study will be confirmed by the DSMC and TSC. This is a rapidly evolving disease area and information about the control rate for progression

© Copyright: The University of Oxford and Oxford University Hospitals NHS Foundation Trust 2019

to hospitalisation or death is not yet fully known. More details will be available during the trial and will be assessed to refine the sample size. Initial assumptions are described below.

The total sample size of 800 patients is based on the following assumptions: 30-40% of patients following standard care will progress to hospitalisation or death within 28 days; 90% power, 2-sided 5% significance and a 30-35% reduction in progression or death for patients on Azithromycin (778 participants required to detect a 33.3% reduction from 30% to 20% in progression to hospitalisation or death or 646 required to detect a 30% reduction from 40% to 28% in progression to hospitalisation or death). Total sample size to be refined at the interim analysis if progression to the full trial is the option chosen.

12.4 Analysis Populations

The intention-to-treat (ITT) population is defined as all randomised patients analysed according to their randomised allocation.

A supplementary ITT population (ITT +ve) is defined as all randomised patients with a positive COVID PCR result.

All efficacy and safety analyses will be based on the ITT population and repeated on the ITT +ve population.

12.5 Stopping Rules

A Data and Safety Monitoring Committee will be set up to review recruitment, trial conduct, safety and efficacy data.

The first formal interim analysis will take place after 100 patients have completed the trial (i.e. approximately 28 days after the 100th patient has been randomised). The DSMC will review the safety of participants and will review the results of the futility analysis (i.e. no evidence of sufficient clinical efficacy to reasonably justify continuing the trial) in order to make recommendations about continuation or otherwise to the full trial. This will be based on Bayesian predictive probabilities, the full details of which will be described in the SAP. If the decision is to continue, then a review of the endpoints and the assumptions taken for the sample size will be undertaken, blinded to treatment allocation, and recommendations provided as to the final sample size for the full trial. Further interim analyses for futility using the same method will be undertaken at the discretion of the DSMC.

© Copyright: The University of Oxford and Oxford University Hospitals NHS Foundation Trust 2019

12.6 The Level of Statistical Significance

All tests will be completed at a 5% 2-sided significance level. All comparative outcomes will be presented as summary statistics with 95% confidence intervals and reported in accordance with the CONSORT Statement (http://www.consort-statement.org).

12.7 Procedure for Accounting for Missing, Unused, and Spurious Data.

Missing data will be minimised by careful data management. Missing data will be described with reasons given where available; the number and percentage of individuals in the missing category will be presented by treatment arm. All data collected on data collection forms will be used, since only essential data items will be collected. No data will be considered spurious in the analysis since all data will be checked and cleaned before analysis.

The nature and mechanism for missing variables and outcomes will be investigated, and if appropriate multiple imputation will be used. In this situation sensitivity analyses will be undertaken assessing the underlying missing data assumptions. Any imputation techniques will be fully described in the Statistical Analysis Plan.

12.8 Procedures for Reporting any Deviation(s) from the Original Statistical Plan

A detailed statistical analysis plan will be drawn up early in the trial with review and appropriate sign-off following OCTRU SOPs. Any changes to the statistical analysis plan during the trial will be subject to the same review and sign-off procedure with details of changes being included in the new version. Any changes/deviations from the original SAP will be described and justified in protocol and/or in the final report, as appropriate.

13 DATA MANAGEMENT

The plan for the data management of the study are outlined below.

13.1 Source Data

Source documents are where data are first recorded, and from which participants' CRF data are obtained. These include, but are not limited to, hospital records (from which medical history and previous and concurrent medication may be summarised into the CRF), clinical and office charts, laboratory and pharmacy records, and radiographs. The study will have a data management plan. All documents will be stored safely in confidential conditions.

13.2 Access to Data

Direct access will be granted to authorised representatives from the Sponsor, host institution and the regulatory authorities to permit trial-related monitoring, audits and inspections.

© Copyright: The University of Oxford and Oxford University Hospitals NHS Foundation Trust 2019

13.3 Data Recording and Record Keeping

The Investigators will maintain appropriate medical and research records for this trial, in compliance with the principles of GCP and regulatory and institutional requirements for the protection of confidentiality of volunteers. The Chief Investigator, site teams and central study team will have access to records. The Investigators will permit authorised representatives of the Sponsor, as well as ethical and regulatory agencies to examine (and when required by applicable law, to copy) clinical records for the purposes of quality assurance reviews, audits and evaluation of the study safety and progress.

All study data will be captured directly in the study's instance of RedCap or the study instance of their randomisation system RRAMP. There are no paper CRFs, worksheets, questionnaires that will be completed as part of this study.

Identifiable information will be recorded on a secure web-based form in the study randomisation system (RRAMP) by the attending clinician or delegate including a member of research team to enable follow-up:

- Patient details e.g. name, NHS (or CHI for Scottish patients) number, date of birth, sex, telephone number and GP details

Note: These data fields will allow sites to check their local hospital records and NHS Spine or other devolved nation systems to check when to contact a patient that they have not deceased or been admitted to avoid any upset of patient's relatives. The GP details are required to allow the central trial co-ordinating team to ensure a letter is sent out to the patient's GP informing them of their participation in the ATOMIC2 study.

- *An email address will also be recorded to enable a copy of the completed consent form to be sent to the patient or at their request a different individual for safe keeping.*

The Investigator and/or Sponsor must retain copies of the essential documents for a minimum of 5 years following the end of the study. Site investigators will always have contemporaneous access to all data entered into the system for patients from their site.

The Investigator will inform the Sponsor of the storage location of the essential documents and of any changes in the storage location should they occur. The Investigator must contact the Sponsor for approval before disposing of any documentation. The Investigator should take measures to prevent accidental or premature destruction of these documents.

13.4 Collection of data

Data will be collected by a member of the clinical or study team. Data will also be collected from ePR/medical notes and NHS Spine and by telephone call.

© Copyright: The University of Oxford and Oxford University Hospitals NHS Foundation Trust 2019

After day 28 of the study, sites will be asked to conduct a notes review to check for any hospital admissions.

14 QUALITY ASSURANCE PROCEDURES

14.1 Risk assessment

A risk assessment will be conducted according to OCTRU's process and a monitoring plan will be drafted to include all central monitoring activities. The trial will be conducted in accordance with the current approved protocol, Principles of GCP, relevant regulations and OCTRU standard operating procedures.

14.2 Monitoring

Due to the nature of this study and timelines and in accordance with the risk assessment in section 14.1 monitoring will be limited to central monitoring activities – there will be no site monitoring, missing data will be queried with sites where mandatory. Monitoring of the data will occur as the data is being entered into the database.

14.3 Quality assurance

The Sponsor or its designated representative will assess each study site to verify the qualifications of each Investigator and the site staff and to ensure that the site has all of the required equipment. A virtual study Initiation meeting will occur where among other things the Investigator will be informed of their responsibilities and procedures for ensuring adequate and correct study documentation.

14.4 Trial committees

14.4.1 Data Safety Monitoring Committee (DSMC)

The DSMC is a group of independent experts external to the trial who assess the progress, conduct, participant safety and critical endpoints of the study. The study DSMC will adopt a DAMOCLES charter which defines its terms of reference and operation in relation to the oversight of the trial. They will review the interim analysis after 100 participants have completed the trial and make recommendations to the TSC as to the continuation or otherwise of the trial. They will also review the endpoints and sample size assumptions to finalise the sample size for the full definitive trial.

© Copyright: The University of Oxford and Oxford University Hospitals NHS Foundation Trust 2019

This Group will provide advice and recommendations to the TSC and may correspond directly with the Sponsor if potential safety concerns are raised.

14.4.2 Trial Steering Committee (TSC)

The TSC include independent members and members of the research team and provides overall supervision of the trial on behalf of the funder. Its terms of reference will be agreed and will be recorded in a TSC charter.

15 PROTOCOL DEVIATIONS

A trial related deviation is a departure from the ethically and regulatory approved trial protocol or other trial document or process (e.g. consent process or IMP administration) or from Good Clinical Practice (GCP) or any applicable regulatory requirements. Any deviations from the protocol will be documented in a protocol deviation form, discussed as per the deviation SOP and filed in the trial master file.

16 SERIOUS BREACHES

The Medicines for Human Use (Clinical Trials) Regulations contain a requirement for the notification of "serious breaches" to the MHRA within 7 days of the Sponsor becoming aware of the breach.

A serious breach is defined as "A breach of GCP or the trial protocol which is likely to affect to a significant degree –

(a) the safety or physical or mental integrity of the subjects of the trial; or

(b) the scientific value of the trial".

In the event that a serious breach is suspected the Sponsor must be contacted within 1 working day. In collaboration with the CI the serious breach will be reviewed by the Sponsor and, if appropriate, the Sponsor will report it to the REC committee, Regulatory authority and the relevant NHS host organisation within seven calendar days.

© Copyright: The University of Oxford and Oxford University Hospitals NHS Foundation Trust 2019

17 ETHICAL AND REGULATORY CONSIDERATIONS

17.1 Declaration of Helsinki

The Investigator will ensure that this trial is conducted in accordance with the principles of the Declaration of Helsinki.

17.2 Guidelines for Good Clinical Practice

The Investigator will ensure that this trial is conducted in accordance with the principles of Good Clinical Practice.

17.3 Approvals

The protocol, informed consent form, participant information sheet and any proposed advertising material will be submitted to an appropriate Research Ethics Committee (REC), HRA, regulatory authorities (MHRA in the UK), and host institution(s) for written approval.

The Investigator will submit and, where necessary, obtain approval from the above parties for all substantial amendments to the original approved documents.

17.4 Reporting

The CI shall submit once a year throughout the clinical trial, or on request, an Annual Progress Report to the REC, HRA, host organisation, funder (where required) and Sponsor, and a DSUR to the MHRA. In addition, an End of Trial notification and final report will be submitted to the MHRA, the REC, host organisation and Sponsor.

17.5 Transparency in Research

Prior to the recruitment of the first participant, the trial will have been registered on a publicly accessible database.

Results will be uploaded to the European Clinical Trial (EudraCT) Database within 12 months of the end of trial declaration by the CI or their delegate.

Where the trial has been registered on multiple public platforms, the trial information will be kept up to date during the trial, and the CI or their delegate will upload results to all those public registries within 12 months of the end of the trial declaration.

17.6 Participant Confidentiality

The study will comply with the General Data Protection Regulation (GDPR) and Data Protection Act 2018, which require data to be de-identified as soon as it is practical to do so. The processing

© Copyright: The University of Oxford and Oxford University Hospitals NHS Foundation Trust 2019

of the personal data of participants will be minimised by making use of a unique participant study number only on all study documents and any electronic database(s), with the exception of the storage of patient and their GP contact details to enable follow-up of the participants. This data is stored in an encrypted form. All documents will be stored securely and only accessible by study staff and authorised personnel. The study staff will safeguard the privacy of participants' personal data.

Instances of missing, discrepant, or uninterpretable data will be queried with the Investigator for resolution. Any changes to study data will be documented in an audit trail, which will be maintained within the clinical database.

In compliance with the principles of ICH GCP and regulatory requirements, the Sponsor, a third party on behalf of the Sponsor, regulatory agencies or Independent Ethics Committees (IEC) may conduct quality assurance audits at any time during or following a study. In the event of monitoring, the Investigator must agree to allow monitoring of the study according to ICH GCP requirements and The Medicines for Human Use (Clinical Trials) Regulations 2004 (including all modifications [Statutory Instruments] made since 2004).

The Investigator should also agree to allow auditors direct access to all study-related documents including source documents. They must also agree to allocate their time and the time of their study staff to the auditors in order to discuss findings and issues.

17.7 CTU Involvement

This study will be coordinated by the UKCRC registered Oxford Clinical Trials Research Unit (OCTRU) at the University of Oxford.

18 FINANCE AND INSURANCE

18.1 Funding

This study is supported by the Oxford Respiratory NIHR Biomedical Research Centre and University of Oxford MSD COVID-19 Research Response Fund, and through funding of a BRC clinical fellow and through an NIHR Senior Research Fellowship to the CI. The CI salary is funded by the Wellcome Trust. The trial drug is provided at no cost by Pfizer Inc; but if it is no longer possible to source the drug from Pfizer any brand can be used. Pfizer Inc has also provided a grant to the study team to enable this study.

© Copyright: The University of Oxford and Oxford University Hospitals NHS Foundation Trust 2019

18.2 Insurance

The University has a specialist insurance policy in place which would operate in the event of any participant suffering harm as a result of their involvement in the research (Newline Underwriting Management Ltd, at Lloyd's of London). NHS indemnity operates in respect of the clinical treatment that is provided.

18.3 Contractual arrangements

Appropriate contractual arrangements will be put in place with all third parties.

19 PUBLICATION POLICY

Publications will acknowledge the funders with the following text: The research was funded by the Wellcome Trust (104553/z/14/z, 211050/Z/18/z),the National Institute for Health Research (NIHR) Oxford Biomedical Research Centre (BRC), the University of Oxford and Pfizer Inc. The study drug is initially being provided free-of-charge by Pfizer Inc. who had no part in the study design, conduct or analysis.

The following disclaimer after the acknowledgement must be added: "The views expressed are those of the author(s) and not necessarily those of the NHS, the NIHR or the Department of Health, the University of Oxford or Pfizer Inc."

20 DEVELOPMENT OF A NEW PRODUCT/ PROCESS OR THE GENERATION OF INTELLECTUAL PROPERTY

Not applicable.

21 ARCHIVING

Both paper and electronic trial data will be retained through an archiving service as per the sponsoring institute's policy, and data will be retained for a minimum of 5 years after termination of the trial.

© Copyright: The University of Oxford and Oxford University Hospitals NHS Foundation Trust 2019

22 REFERENCES

1. Arabi YM, Deeb AM, Al-Hameed F, et al. Macrolides in critically ill patients with Middle East Respiratory Syndrome. Int J Infect Dis 2019;81:184-90.
2. Gautret P, Lagier J, Parola P, et al. Hydroxychloroquine and azithromycin as a treatment of COVID-19: results of an open-label non-randomized clincial trial. Int J of Antimicrobial Agents 2020.
3. Belhadi D, Peiffer-Smadja N, Yazdanpanah Y, Mentre F, Laouenan C. A brief review of antiviral drugs evaluated in registered clinical trials for COVID-19. medRxiv 2020.
4. Gielen V, Johnston SL, Edwards MR. Azithromycin induces anti-viral responses in bronchial epithelial cells. Eur Respir J 2010;36:646-54.
5. Schogler A, Kopf BS, Edwards MR, et al. Novel antiviral properties of azithromycin in cystic fibrosis airway epithelial cells. Eur Respir J 2015;45:428-39.
6. Porter JD, Watson J, Roberts LR, et al. Identification of novel macrolides with antibacterial, anti-inflammatory and type I and III IFN-augmenting activity in airway epithelium. The Journal of antimicrobial chemotherapy 2016;71:2767-81.
7. Gibson PG, Yang IA, Upham JW, et al. Effect of azithromycin on asthma exacerbations and quality of life in adults with persistent uncontrolled asthma (AMAZES): a randomised, double-blind, placebo-controlled trial. Lancet 2017;390:659-68.
8. Johnston SL, Pattemore PK, Sanderson G, et al. Community study of role of viral infections in exacerbations of asthma in 9-11 year old children. BMJ 1995;310:1225-9.
9. Johnston SL, Pattemore PK, Sanderson G, et al. The relationship between upper respiratory infections and hospital admissions for asthma: a time-trend analysis. Am J Respir Crit Care Med 1996;154:654-60.
10. Retallack H, Di Lullo E, Arias C, et al. Zika virus cell tropism in the developing human brain and inhibition by azithromycin. Proc Natl Acad Sci U S A 2016;113:14408-13.
11. Li C, Zu S, Deng YQ, et al. Azithromycin Protects against Zika virus Infection by Upregulating virus-induced Type I and III Interferon Responses. Antimicrob Agents Chemother 2019.
12. Zeng S, Meng X, Huang Q, et al. Spiramycin and azithromycin, safe for administration to children, exert antiviral activity against enterovirus A71 in vitro and in vivo. Int J Antimicrob Agents 2019;53:362-9.
13. Madrid PB, Panchal RG, Warren TK, et al. Evaluation of Ebola Virus Inhibitors for Drug Repurposing. ACS Infect Dis 2015;1:317-26.
14. Kouznetsova J, Sun W, Martinez-Romero C, et al. Identification of 53 compounds that block Ebola virus-like particle entry via a repurposing screen of approved drugs. Emerg Microbes Infect 2014;3:e84.
15. Kawamura K, Ichikado K, Takaki M, Eguchi Y, Anan K, Suga M. Adjunctive therapy with azithromycin for moderate and severe acute respiratory distress syndrome: a retrospective, propensity score-matching analysis of prospectively collected data at a single center. Int J Antimicrob Agents 2018;51:918-24.
16. Lee N, Wong CK, Chan MCW, et al. Anti-inflammatory effects of adjunctive macrolide treatment in adults hospitalized with influenza: A randomized controlled trial. Antiviral Res 2017;144:48-56.
17. Cao B, Wang Y, Wen D, et al. A Trial of Lopinavir-Ritonavir in Adults Hospitalized with Severe Covid-19. N Engl J Med 2020.

© Copyright: The University of Oxford and Oxford University Hospitals NHS Foundation Trust 2019

18. Baden LR, Rubin EJ. Covid-19 - The Search for Effective Therapy. N Engl J Med 2020.
19. Martinez MA. Compounds with therapeutic potential against novel respiratory 2019 coronavirus. Antimicrob Agents Chemother 2020.
20. Ruan Q, Yang K, Wang W, Jiang L, Song J. Clinical predictors of mortality due to COVID-19 based on an analysis of data of 150 patients from Wuhan, China. Intensive care medicine 2020.
21. Huang C, Wang Y, Li X, et al. Clinical features of patients infected with 2019 novel coronavirus in Wuhan, China. Lancet 2020;395:497-506.
22. Shakoory B, Carcillo JA, Chatham WW, et al. Interleukin-1 Receptor Blockade Is Associated With Reduced Mortality in Sepsis Patients With Features of Macrophage Activation Syndrome: Reanalysis of a Prior Phase III Trial. Crit Care Med 2016;44:275-81.
23. Xu Z, Shi L, Wang Y, et al. Pathological findings of COVID-19 associated with acute respiratory distress syndrome. Lancet Respir Med 2020.
24. Wong CK, Lam CW, Wu AK, et al. Plasma inflammatory cytokines and chemokines in severe acute respiratory syndrome. Clin Exp Immunol 2004;136:95-103.
25. Mahallawi WH, Khabour OF, Zhang Q, Makhdoum HM, Suliman BA. MERS-CoV infection in humans is associated with a pro-inflammatory Th1 and Th17 cytokine profile. Cytokine 2018;104:8-13.
26. Altenburg J, de Graaff CS, van der Werf TS, Boersma WG. Immunomodulatory effects of macrolide antibiotics - part 1: biological mechanisms. Respiration 2011;81:67-74.
27. Parnham MJ, Erakovic Haber V, Giamarellos-Bourboulis EJ, Perletti G, Verleden GM, Vos R. Azithromycin: mechanisms of action and their relevance for clinical applications. Pharmacology & therapeutics 2014;143:225-45.
28. Marjanovic N, Bosnar M, Michielin F, et al. Macrolide antibiotics broadly and distinctively inhibit cytokine and chemokine production by COPD sputum cells in vitro. Pharmacol Res 2011;63:389-97.
29. Shinkai M, Foster GH, Rubin BK. Macrolide antibiotics modulate ERK phosphorylation and IL-8 and GM-CSF production by human bronchial epithelial cells. Am J Physiol Lung Cell Mol Physiol 2006;290:L75-85.
30. Hui D, Yan F, Chen RH. The effects of azithromycin on patients with diffuse panbronchiolitis: a retrospective study of 29 cases. J Thorac Dis 2013;5:613-7.
31. Kudoh S, Azuma A, Yamamoto M, Izumi T, Ando M. Improvement of survival in patients with diffuse panbronchiolitis treated with low-dose erythromycin. Am J Respir Crit Care Med 1998;157:1829-32.
32. Nagai H, Shishido H, Yoneda R, Yamaguchi E, Tamura A, Kurashima A. Long-term low-dose administration of erythromycin to patients with diffuse panbronchiolitis. Respiration 1991;58:145-9.
33. Weng D, Wu Q, Chen XQ, et al. Azithromycin treats diffuse panbronchiolitis by targeting T cells via inhibition of mTOR pathway. Biomed Pharmacother 2019;110:440-8.
34. Zhou Y, Fu B, Zheng X, et al. Aberrant pathogenic GM-CSF+ T cells and inflammatory CD14+CD16+ monocytes in severe pulmonary syndrome patients of a new coronavirus. bioRxiv 2020.
35. Liao M, Liu Y, Yuan J, et al. The landscape of lung bronchoalveolar immune cells in COVID-19 revealed by
single-cell RNA sequencing bioRxiv 2020.

© Copyright: The University of Oxford and Oxford University Hospitals NHS Foundation Trust 2019

36. Bosnar M, Cuzic S, Bosnjak B, et al. Azithromycin inhibits macrophage interleukin-1beta production through inhibition of activator protein-1 in lipopolysaccharide-induced murine pulmonary neutrophilia. Int Immunopharmacol 2011;11:424-34.
37. Meyer M, Huaux F, Gavilanes X, et al. Azithromycin reduces exaggerated cytokine production by M1 alveolar macrophages in cystic fibrosis. Am J Respir Cell Mol Biol 2009;41:590-602.
38. Banjanac M, Munic Kos V, Nujic K, et al. Anti-inflammatory mechanism of action of azithromycin in LPS-stimulated J774A.1 cells. Pharmacol Res 2012;66:357-62.
39. Khan AA, Slifer TR, Araujo FG, Remington JS. Effect of clarithromycin and azithromycin on production of cytokines by human monocytes. Int J Antimicrob Agents 1999;11:121-32.
40. Hodge S, Hodge G, Jersmann H, et al. Azithromycin improves macrophage phagocytic function and expression of mannose receptor in chronic obstructive pulmonary disease. Am J Respir Crit Care Med 2008;178:139-48.
41. Murphy BS, Sundareshan V, Cory TJ, Hayes D, Jr., Anstead MI, Feola DJ. Azithromycin alters macrophage phenotype. The Journal of antimicrobial chemotherapy 2008;61:554-60.
42. Yamauchi K, Shibata Y, Kimura T, et al. Azithromycin suppresses interleukin-12p40 expression in lipopolysaccharide and interferon-gamma stimulated macrophages. Int J Biol Sci 2009;5:667-78.
43. Legssyer R, Huaux F, Lebacq J, et al. Azithromycin reduces spontaneous and induced inflammation in DeltaF508 cystic fibrosis mice. Respir Res 2006;7:134.
44. Press BGP. The British National Formulary. London, UK2020.
45. Milberg P, Eckardt L, Bruns HJ, et al. Divergent proarrhythmic potential of macrolide antibiotics despite similar QT prolongation: fast phase 3 repolarization prevents early afterdepolarizations and torsade de pointes. J Pharmacol Exp Ther 2002;303:218-25.
46. Kezerashvili A, Khattak H, Barsky A, Nazari R, Fisher JD. Azithromycin as a cause of QT-interval prolongation and torsade de pointes in the absence of other known precipitating factors. J Interv Card Electrophysiol 2007;18:243-6.
47. Kanoh S, Rubin BK. Mechanisms of action and clinical application of macrolides as immunomodulatory medications. Clin Microbiol Rev 2010;23:590-615.
48. Ray WA, Murray KT, Hall K, Arbogast PG, Stein CM. Azithromycin and the risk of cardiovascular death. N Engl J Med 2012;366:1881-90.
49. Cheng YJ, Nie XY, Chen XM, et al. The Role of Macrolide Antibiotics in Increasing Cardiovascular Risk. J Am Coll Cardiol 2015;66:2173-84.
50. Polgreen LA, Riedle BN, Cavanaugh JE, et al. Estimated Cardiac Risk Associated With Macrolides and Fluoroquinolones Decreases Substantially When Adjusting for Patient Characteristics and Comorbidities. J Am Heart Assoc 2018;7.
51. Hansen MP, Scott AM, McCullough A, et al. Adverse events in people taking macrolide antibiotics versus placebo for any indication. Cochrane Database Syst Rev 2019;1:CD011825.
52. Zhang C, Shi L, Wang FS. Liver injury in COVID-19: management and challenges. Lancet Gastroenterol Hepatol 2020.
53. Bangash MN, Patel J, Parekh D. COVID-19 and the liver: little cause for concern. Lancet Gastroenterol Hepatol 2020.
54. Limited S. Summary of Product Characteristics 2019 25/09/2019
55. Severity Grading In Drug Induced Liver Injury. LiverTox: Clinical and Research Information on Drug-Induced Liver Injury. Bethesda (MD)2012.

© Copyright: The University of Oxford and Oxford University Hospitals NHS Foundation Trust 2019

56. Bahat Dinur A, Koren G, Matok I, et al. Fetal safety of macrolides. Antimicrob Agents Chemother 2013;57:3307-11.
57. LAB-0606-6.0. 2019. (Accessed 30/04/2020, 2020, at http://labeling.pfizer.com/ShowLabeling.aspx?id=650#Data1.)

© Copyright: The University of Oxford and Oxford University Hospitals NHS Foundation Trust 2019

23 APPENDIX A: Figure 1 – Background data for trial rationale

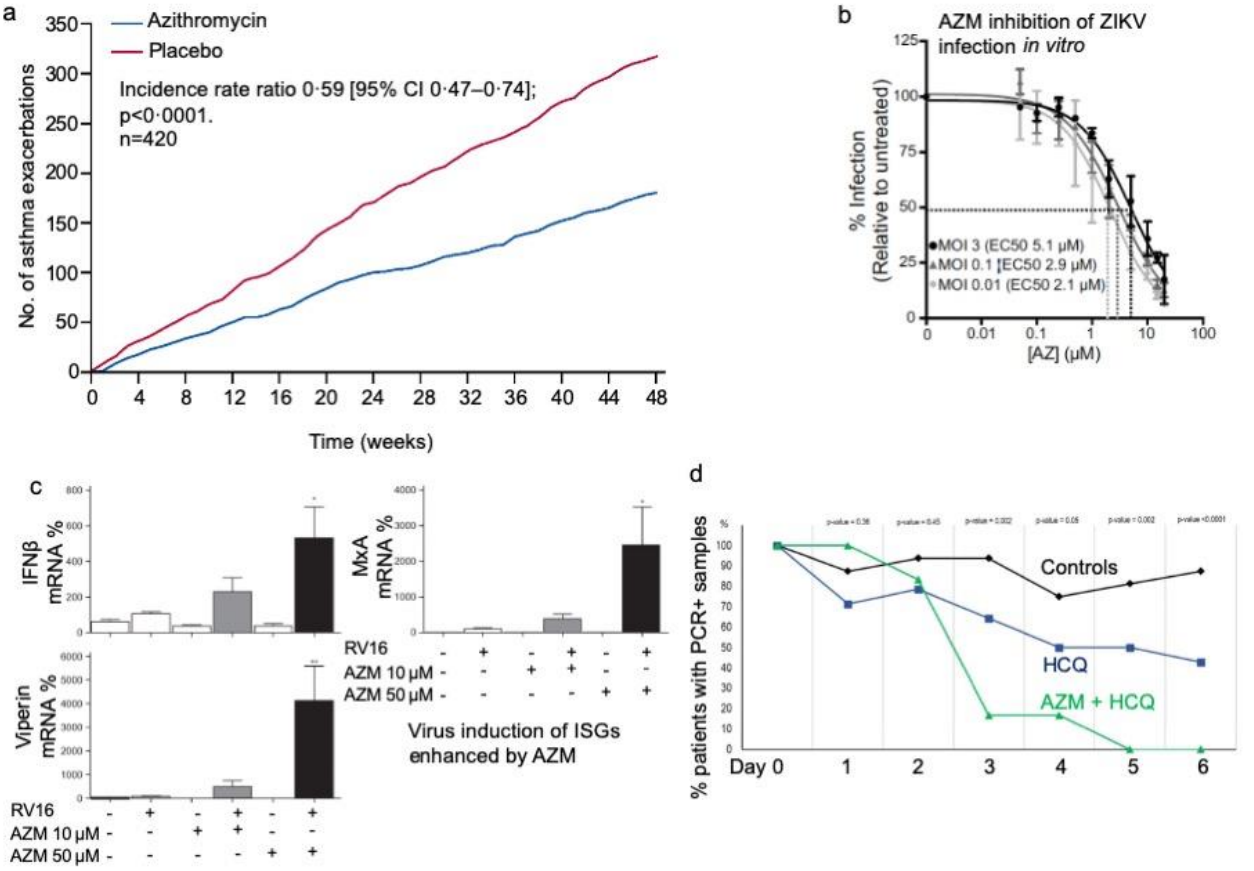

Figure 1 (a) Cumulative severe and moderate asthma exacerbations during 48 weeks of treatment with Azithromycin 500 mg, three times per week, or placebo in AMAZES[7] (b) Azithromycin inhibits ZIKV infection in U87 glial cells. EC_{50} values for AZM-mediated reduction of ZIKV infection were 5.1 µM, 2.9 µM, 2.1 µM for an MOI of 3, 0.1, 0.01 respectively[10]. (c) AZM enhanced rhinovirus-induced interferon-stimulated genes in human bronchial epithelial cells[4]. (d) PCR + status in small, non-randomised clinical trial suggesting earlier return to PCR-ve status with combined hydroxychloroquine (HCQ) and Azithromycin (AZM)[2].

© Copyright: The University of Oxford and Oxford University Hospitals NHS Foundation Trust 2019

24 APPENDIX B: SCHEDULE OF PROCEDURES

	Study day			
**Procedures**	Day 0 (Day of randomisation)	14 days after randomisation (Study Day 14) – Participant contacted by phone	28 days after randomisation (Study Day 28) – Participant contacted by phone	Any hospital admission
Consent	✓			
Eligibility check	✓			
Demographics	✓			
Medical history	✓			
Medication history	✓	✓	✓	✓
Swab taken for COVID PCR test (if possible)	✓			
Randomisation	✓			
Dispensing of 14 day course of IMP (if randomised to IMP)	✓			
Medical notes / ePR / biochemistry results/ microbiology results review	✓			✓
Radiology review (if any performed on clinical grounds)	✓			✓
Assessment of outcome measures (vital status, history of admission)(ePR/ notes / Death register / Telephone call)		✓	✓	✓
Compliance assessment (telephone call)		✓	✓	
Study Blood sampling (optional)	✓ (serum sample + Tempus, EDTA)			✓
Nasal brush (optional, for observational)	✓			
SAE/AE reporting	✓	✓	✓	✓

CONFIDENTIAL Clinical Trial Protocol Template version 15.0
© Copyright: The University of Oxford and Oxford University Hospitals NHS Foundation Trust 2019

25 APPENDIX C: EXAMPLE OF PARTICIPANT'S STUDY JOURNEY

Study Day	Day of the Week	Study procedures
0	Monday	Recruitment<br>Consent<br>Randomisation<br>First dose of IMP (if allocated)
1	Tuesday	Second dose of IMP (if allocated)
2	Wednesday	Third dose of IMP (if allocated)
3	Thursday	Fourth dose of IMP (if allocated)
4	Friday	Fifth dose of IMP (if allocated)
5	Saturday	Sixth dose of IMP (if allocated)
6	Sunday	Seventh dose of IMP (if allocated)
7	Monday	Eighth dose of IMP (if allocated)
8	Tuesday	Ninth dose of IMP (if allocated)
9	Wednesday	Tenth dose of IMP (if allocated)
10	Thursday	Eleventh dose of IMP (if allocated)
11	Friday	Twelfth dose of IMP (if allocated)
12	Saturday	Thirteenth dose of IMP (if allocated)
13	Sunday	Fourteenth dose of IMP (if allocated) (THIS IS THE LAST DOSE)
14	Monday	Check at site that it is suitable to contact the participant<br><br>Day 14 follow-up of patient by telephone
28	Monday	Check at site that it is suitable to contact the participant<br><br>Day 28 follow-up of patient by telephone

The above assumes that a study participant does not get admitted to hospital nor dies within 28 days of randomisation.

If a participant gets admitted to hospital within 28 days of randomisation – then they are followed up whilst in hospital until they are discharged – this may go past 28 days since randomisation.

If a participant dies before 28 days of randomisation – this will be recorded at recruiting sites using data either held in hospital records or on NHS Spine and there may not be any data collection at day 14 or day 28.

© Copyright: The University of Oxford and Oxford University Hospitals NHS Foundation Trust 2019

26 APPENDIX D: AMENDMENT HISTORY

Amendment No.	Protocol Version No.	Date issued	Author(s) of changes	Details of Changes made
1 (Non substantial)	3.0	07May2020	Timothy Hinks and Susan Dutton	Updated following meeting of TSC/DSMC requesting clarity on analyses to be undertaken.<br><br>Typographical changes made<br>Reduction of number of optional blood tubes to be taken.<br>Inclusion at baseline data collection for females' questions about pregnancy and lactation, and data on any other COVID-19 trials participants enter if admitted to hospital.
	2.0	30Apr2020	Timothy Hinks	Updated with changes requested by the REC, specifically more information on pregnancy and the risks of the trial (section 11.1.6) and some potential drug interactions (section 11.1.5) specifically Coumarin-type oral anticoagulants, Nelfinavir, Trimethoprim/ Sulfamethoxazole.
	1.0	23Apr2020	First issue	

© Copyright: The University of Oxford and Oxford University Hospitals NHS Foundation Trust 2019
