# Supplementary material for: A multi-centre open-label two-arm randomised superiority clinical trial of azithromycin versus usual care in ambulatory COVID-19: study protocol for the ATOMIC2 trial
Source: Trials. 2020 Aug 17;21:718. doi: 10.1186/s13063-020-04593-8 (PMC7429453; doi:10.1186/s13063-020-04593-8)
Supplement: Supplementary file 4 — Additional file 4. [file 13063_2020_4593_MOESM4_ESM.pdf]

Insert local trust logo

A study of an antibiotic called Azithromycin for those who come to hospital with COVID-19 symptoms who are not admitted to hospital (otherwise known as Ambulatory COVID-19) – ATOMIC2

(Full scientific name of the study: A multi-centre open-label two-arm randomised superiority clinical trial of Azithromycin versus usual care in Ambulatory COVID-19)

PATIENT INFORMATION LEAFLET

Version 4.0, 07May2020

Invitation to join the ATOMIC study

We would like to invite you to take part in a research study (also known as a clinical trial).

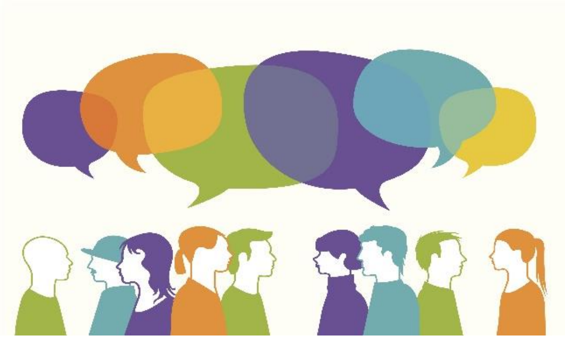

Before you decide whether to take part or not, it is important that you understand why we are doing this study and what it will involve.

Please take time to read the following information and talk to others about the study. If anything is unclear, or if you would like more information, please ask a member of the study team who will be happy to answer any questions.

What is the purpose of this study?

COVID-19 is caused by severe acute respiratory syndrome coronavirus 2 (SARS-CoV-2) infection. Most (8 out of 10) people with the disease have only mild symptoms, but in some people the condition can progress from a mild form of disease with fever and cough, to severe respiratory failure requiring mechanical ventilation over 1 to 2 weeks.

The purpose of this study is to assess whether the use of a commonly given antibiotic called Azithromycin can prevent symptoms in patients, who go to hospital but doctors decide there is no need to admit them for treatment, from getting worse.

What exactly is Azithromycin?

- Azithromycin is an antibiotic which is widely and safely used to treat infections and excessive inflammation in the lung, for conditions such as pneumonia, and infections of the nose and throat such as sinus infection (sinusitis). It is also often used in children to treat chest and ear infections.
- It is a type of antibiotic that fights infection by killing bacteria, and it also has properties that fight viral infections and reduces inflammation.

- These additional anti-viral properties have led to its use in other viral infections similar to COVID-19 but it is yet to be known if it will help patients with COVID-19.

- The important side effects of azithromycin are as follows:

Side Effect	Frequency
Loose stools, nausea (sickness)	Very common (in more than 1 in 10 people taking it)
Dizziness, drowsiness, fatigue, headache, skin rash, abdominal pain, mild abnormalities in liver and kidney function tests, chest pain and palpitations, painful joints	Common (more than 1 in 100 but fewer than 1 in 10 people taking it)
Irregular rhythms of the heart (arrhythmia), kidney or liver failure, ringing in your ears (tinnitus), severe abdominal pain (pancreatitis) and severe allergic reactions (anaphylaxis)	Uncommon (more than 1 in 1000 but fewer than 1 in 100 people taking it)

Who is taking part and why have I been invited to take part?

We are hoping to enrol up to 800 men and women aged 18 or over from at least 4 NHS hospitals in the UK.

You have been invited to take part because as an adult aged 18 years or older who is being assessed at hospital, and your doctor believes you probably have COVID-19 infection, but the doctors feel your symptoms are sufficiently mild that you do not need to be admitted and can be managed as an outpatient at your usual residence.

Do I have to take part in this study?

You are under no obligation to take part in the study. Deciding not to will not affect the treatment/care you receive from your team. It is up to you to decide whether or not to take part. Please keep this leaflet and use it as it may help you make your decision. If you decide to take part, you will be asked to sign a consent form.

If you choose not to join the ATOMIC2 study, you will receive the routine NHS care, as agreed by your local treating team of healthcare professionals, in accordance with standard NHS guidelines. A note will be made of your age and gender, so that we can find out who decides not to take part. You cannot be identified from this data. A researcher may ask you if you would be happy to give a reason for not wanting to take part in the study. Giving this information is entirely voluntary.

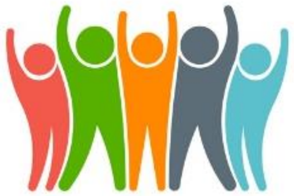

Should you decide to take part, you are still free to withdraw at any time and without giving a reason. This will not affect the standard of care you receive as either an inpatient or an outpatient from the NHS.

What will happen if I take part?

If you are happy to take part in this study, a researcher will ask you some simple questions and check your medical history to confirm that you are eligible.

Initial assessment: If you are eligible, you will be asked to sign and date a consent form for the study. We will then ask you some questions about your medical history and to check you are not already taking any drugs (medications) that Azithromycin should not be taken with and some questions about your health. These questions should take you no more than 10 minutes to answer.

There are no additional tests that you will have as a result of agreeing to take part in this study with one exception. At the time of this leaflet being written, people who come to hospital, where doctors decide not to admit are not routinely having a COVID-19 swab taken to confirm or deny whether the symptoms are as a result of COVID-19, unless there is a reason such as being an essential worker and the need to know whether you are able to return to front line duties. We ask that you agree to having this COVID-19 swab, <u>if the hospital is able to do this</u>, this is like a big cotton wool bud that is placed into your nose and moved around to remove some cells. *Please note that in most cases, we will not be able to tell you the results of this swab if one is taken, as most swabs will not be processed for several weeks, and testing may not be performed within the NHS laboratory systems.*

As part of standard NHS care, you will have an ECG (an electrical tracing of your heart), blood tests and a chest x-ray, as part of your COVID-19 assessment – we will ask for your permission to review and record these results for the study team.

Allocation: You will then be randomly allocated to one of the study groups by a computer, i.e. allocation is by chance. You will have an equal chance of being allocated to either group, like the toss of a coin. The computer would put you either into:

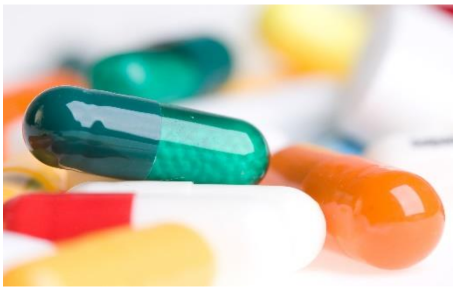

- The Azithromycin group – where we would ask you to take Azithromycin for 14 days. We would ask you to take the capsules (tablets) once a day, about the same time each day – either 1 hour before or 2 hours after a meal. *Please note – you would only be given capsules for 14 days – there would not be any further capsules supplied by the study after you leave the hospital*.
- The Current standard care group – where we would not give you any Azithromycin to take.

There is a 50% chance that you will be in either group, and currently doctors do not know if Azithromycin will make a difference to COVID-19 patients or not. For both groups, the doctors will give you the latest current advice – which may include rest, pain relief and advice on when it might be appropriate to return to hospital if needed. Being part of the ATOMIC2 study will not change the way your local hospital cares for you and the standard treatments they would give you. If you have already been prescribed antibiotics as part of your clinical care, you can continue to take these to complete the course.

The random allocation is important because this way, we can test the different treatments fairly and nobody can influence into which group you are placed. Your healthcare and research teams will not be able to affect which group you are put into and you will not be able to choose. You should note, that is not in NHS guidelines for you to be given Azithromycin for the symptoms of COVID-19, so if you decide not to participate in this trial, or if you agree and are not allocated the Azithromycin, the team looking after you will not be able to give you a prescription for Azithromycin, as this is the reason for this study to see if the drug does cause a difference.

At some sites, if time and resources permit and you are well enough, they may also ask you if in addition to the above COVID-19 swab whether you would be prepared to give some blood for some extra tests. These tests are only for the purposes of this study and the results will not be available to your clinical care team and your GP.

Are there any optional parts of the study?

Yes some blood and a nose fluid samples.

So far, this leaflet has listed all the things that we would ask of everyone taking part in the study. We are also asking people to donate some blood and nose fluid samples but these are not necessary to be part of the study. The samples would be used to help researchers understand more the impact COVID-19 and any drugs you might be given are having on your body.

If you agree to give these samples – we would ask you to give up to 40mls of blood (this is the equivalent of 3 tablespoons) and allow the researchers to take a sample of your nose fluid. This would involve a small brush or sponge being inserted and gently rubbed inside the nostril. This takes a few seconds to do and may cause sneezing and/or coughing. Occasionally there may be a little discomfort and rarely minor bleeding. A small piece of paper will be inserted into the nostril to collect nasal fluid (nose).

All of these samples are entirely optional – and you can decide to give all or none of them. If you do agree to give samples – if you were to be admitted to hospital within 28 days of randomisation – we might also take 32ml of blood (this is the equivalent of just over 2 tablespoons) and another collection of some nasal fluid if you agree on your consent form to this.
To help keep your information confidential, your sample and any information recorded about you in this study will be 'de-identified' and assigned a study code. However, your DNA is unique to you so it can never be completely anonymous. Commercial organisations might be involved in analysis of the samples.

NOTE – TO TAKE PART IN THE STUDY YOU DO NOT NEED TO AGREE TO GIVE ANY SAMPLES – OTHER THAN A COVID-19 SWAB – EVERYTHING ELSE IS ENTIRELY OPTIONAL

The samples would be stored at your local hospital and at the end of the study, the samples would be transported to the University of Oxford. Your de-identified samples would then be used mainly by local researchers (if applicable), but ethically approved research projects may take place in hospitals, universities, non-profit institutions or commercial laboratories worldwide.

What happens after I leave the hospital today?

If you take part in the study, we would ask you to take the capsules (tablets) if you are allocated to the Azithromycin group for 14 days. You will be given the capsules today at the hospital, you would not need to go to a chemist to pick them up. For all patients, we would like to contact you 14 and 28 days after agreeing to take part to see how you are. This contact would be by telephone. We would ask you some questions about your symptoms, how you have been finding taking the Azithromycin if you are in that group, if you have been

given any new drugs from your GP and give you the opportunity to ask questions, the call should take no more than 10 minutes to answer.

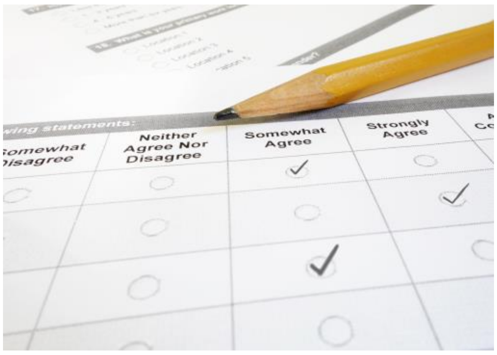

If you are admitted to hospital over the next 28 days, we would ask your permission to access your hospital medical notes for your stay in hospital. Members of the research team may then take the results of any clinical tests, X-rays and observations taken. There would be no additional tests requested by the research team.

If you are taking an oral coumarin anticoagulant such as warfarin and are prescribed azithromycin or any other antibiotic we recommend you attend your GP surgery for a repeat INR check after 3 to 5 days to check this has not affected your INR levels.

For those that take part in the study, your NHS care will not change. Your time in the assessment unit you are being seen in will not change – no additional procedures will be undertaken for the purposes of this study.

What are the benefits and risks of taking part in the study?

The information from this study we hope will answer the question of whether Azithromycin is a drug that should be given to or not given to patients who come to hospitals or GP surgeries who do not need to be admitted to hospital. We cannot promise the study will help you directly, but the information we get has the potential to be of benefit to those who start to show COVID-19 symptoms.

Azithromycin has been widely used in people who are pregnant without evidence of harm to mother or baby. If you are pregnant you may still take part in the study but you may wish to discuss the risks and benefits of this with the study team. Breastfeeding is not recommended until 2 days after discontinuation of azithromycin.

People sometimes feel uncomfortable answering certain questions about their health, or may be unable to answer. If you feel uncomfortable at any point, then you do not have to answer the questions.

Who will know that I am taking part?

The only people who will know that you are taking part in this study are the members of the research team and the healthcare professionals involved in your care. You can tell anyone you would like to that you are taking part.

The only people who will have access to information that identifies you will be people who need to contact you to about the study, or review your hospital record, or the central study team who will let your GP know if you agree to take part in the study, this may include staff from the University of Oxford, the NHS Trust where you were recruited and a specialist group of research nurses employed by a national research support organisation called the Clinical Research Network. The people who analyse the information will not be able to identify you and will not be able to find out your name or contact details. Representatives from the sponsor and << Insert local Trust >> may also need access to monitor the study. Paperwork that is completed by the research

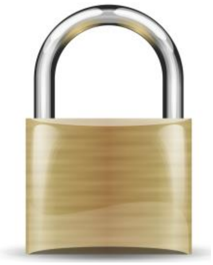

team, or the treating clinical team, will be uploaded onto an electronic database managed by the University of Oxford.

We will contact your GP (doctor) to tell them that you have agreed to take part in the ATOMIC-2 study and whether you were allocated to 14 days of Azithromycin capsules. However, we will not share with them anything you answer to the study team.

Will my details be kept confidential?

The research team will keep all the information collected about and from you confidential. It will not be available to people outside of the research team. The responses you give at day 14 and day 28 – a copy of this data will be given to the hospital you were treated at.

At the end of the study, all of the data will be de-identified so that no-one can be identified. With your permission, this de-identified data will be shared so that more researchers can gain a deeper understanding about patients who have had COVID-19. This information will not identify you, and will not be combined with other information in a way that could identify you. The information will only be used for the purpose of healthcare research, and cannot be used to contact you, nor will it affect your care. In line with what happens in the NHS, the only situation that confidentiality would need to be broken would be if you told a health professional or research team member of something that could result in harm to yourself or others.

What will happen to my data?

Data protection regulation requires that we state the legal basis for processing information about you. In the case of research, this is 'a task in the public interest.' The University of Oxford is the data controller and is responsible for looking after your information as part of ATOMIC-2, and using it properly, anonymised data may get shared with the study funders. We will be using information from you and your medical records, NHS spine (or the equivalent system in Scotland, if you are recruited from Scotland), in order to undertake this study and will use the minimum personally-identifiable information possible. We will keep identifiable information about you for 1 year after the study has finished. This excludes any research documents with personal information, such as consent forms, which will be held securely at the University of Oxford for 5 years after the end of the study. If you agree to your samples being used in future research, your consent form will be held until the samples have been depleted or destroyed.

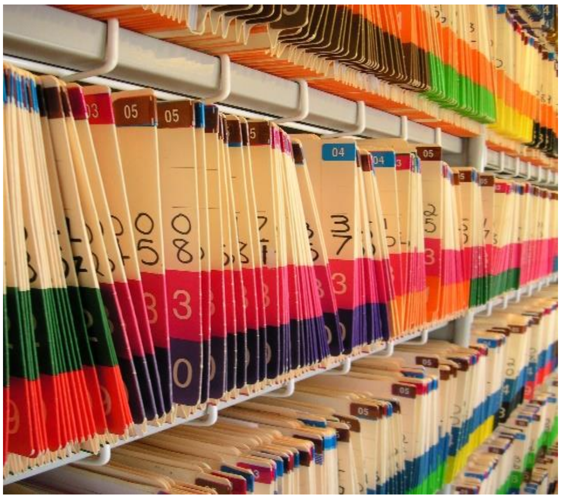

The [local NHS Trust name here] will use your contact details to get in touch with you. They will keep your identifiable information safely for 1 year after the study has finished. Consent forms and study documents held at [local NHS Trust name] will be archived securely, in accordance with their local procedures.

Data protection regulation provides you with control over your personal data and how it is used. When you agree to your information being used in research, however, some of those rights may be limited in order for the research to be reliable and accurate. Further information about your rights with respect to your personal data is available at https://compliance.web.ox.ac.uk/individual-rights

You can find out more about how we use your information by contacting the ATOMIC-2 study team on: atomic2@ndorms.ox.ac.uk

What will happen if I don't want to carry on with the study?

You are free to withdraw from taking part in the study at any time without giving a reason. Please remember, it is your decision to take part. If you agree to take part now, but you change your mind during the study, this will not change the standard of care you receive from the NHS. If you were to decide to stop taking part in the study at any time, any data collected on you would be kept, unless you specify that you want all your data removed. You would not be contacted about the study again or have any further data collected.

What happens at the end of the study?

We will share the results with healthcare researchers and professionals to improve future COVID-19 patient care. Also, we will present them in research reports, at scientific conferences, and publish them in scientific journals. The study results will also be publically available at www.atomic2.octru.ox.ac.uk at the end of the study.

We will not include any data that could identify you in the results. If any of the funders of this research ask us to make the study data available for other researchers or to themselves, we will first make your information anonymous (ie. we will take your name and other identifying details out) so that you cannot be identified.

Who is organising and funding the research?

The University of Oxford is organising this study. It is being conducted by a research team led by Dr Timothy Hinks, Consultant Respiratory Physician at the Oxford University Hospitals NHS Foundation Trust and the University of Oxford. The trial is also being supported by a Nationally Accredited Clinical Trial Unit called the Oxford Clinical Trials Research Unit.

The study has been funded by multiple charities who are supporting COVID-19 studies, at the time of writing this information leaflet this includes the NIHR Biomedical Research Centres and the University of Oxford. Pfizer Inc has also provided the medication and additional funding to the study team to enable this study to be conducted.

Who has approved this study?

A panel of independent researchers and patient representatives, as well as a Research Ethics Committee have reviewed and approved this study.

What if I have concerns?

The University of Oxford, as the study sponsor, has appropriate insurance in place in the unlikely event that you suffer any harm as a direct consequence of your participation in this study. If you have any concerns or complaints about any aspect of the study, please contact the ATOMIC2 research team using the details below. You can also contact the University of Oxford Clinical Trials and Research Governance office on 01865 616480 or by email on ctrg@admin.ox.ac.uk

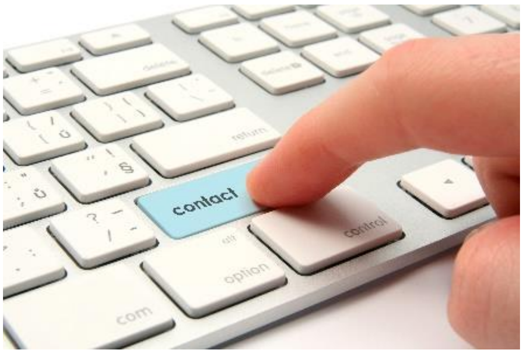

If you would prefer to speak with someone who is not involved in the study, then please contact the Patient Advice and Liaison Service (PALS). PALS is a confidential NHS service that can provide you with support for any complaints or queries you have regarding the care you receive as an NHS patient. However, PALS cannot provide information about this research study.

PALS phone number: <Insert local PALS no>
PALS email: <insert local PALS email>

If you have any questions about the study, please contact the ATOMIC-2 team on:
Email: Atomic2@ndorms.ox.ac.uk Telephone: 01865 223469

Thank you for reading this information leaflet and considering taking part.
