# Supplementary material for: A multi-centre open-label two-arm randomised superiority clinical trial of azithromycin versus usual care in ambulatory COVID-19: study protocol for the ATOMIC2 trial
Source: Trials. 2020 Aug 17;21:718. doi: 10.1186/s13063-020-04593-8 (PMC7429453; doi:10.1186/s13063-020-04593-8)
Supplement: Supplementary file 5 — Additional file 5. [file 13063_2020_4593_MOESM5_ESM.pdf]

PARTICIPANT CONSENT FORM – IRAS ID: 282892

A multi-centre open-label two-arm randomised superiority clinical trial of Azithromycin versus usual care in Ambulatory COVID-19) (ATOMIC2)

If you agree, please initial box

1. I confirm that I have read the information sheet dated 07May2020 (version 4.0) for this study. I have had the opportunity to consider the information, ask questions and have had these answered satisfactorily.	
2. I understand that my participation is voluntary and that I am free to withdraw at any time without giving any reason, without my medical care or legal rights being affected.	
3. I understand that relevant sections of my medical notes and data collected during the study may be looked at by individuals from University of Oxford, from regulatory authorities and from the NHS Trust(s), where it is relevant to my taking part in this research. I give permission for these individuals to have access to my records.	
4. I agree to my General Practitioner (GP) being informed of my participation in this study.	
5. I agree that my de-identified data can be put into a research data repository, where it will remain for 5 years after the end of the study, and may be passed onto any of the funders of this study, including data related to safety.	
6. I agree to my personal contact details being recorded.	
7. I understand that the information held and maintained by NHS Spine or if you are not recruited in England or Wales, the devolved nation equivalent system, may be used to provide information about my health status.	
8. I agree to take part in this study.	

Optional :

	Yes	No
9. I agree to donate blood and nasal brushing samples if time and resources allow. I consider these samples a gift to the University of Oxford and I understand I will not gain any direct personal or financial benefit from them. (This includes samples taken today and if I am admitted to hospital in the next 28 days). I understand this research may involve third parties working with the University of Oxford and also commercial organisations.		
10. I understand and agree that my samples will be used in research aimed at understanding the genetic influences on disease and that the results of these investigations will not be made available to me and are unlikely to have any implications for me personally.	Yes	No
11. I agree for my de-identified samples to be used in future research, here or abroad including the EEA, which has ethics approval. These samples may be shared with companies, you would not receive any payment nor would the company know any of your personal information.	Yes	No

Name of participant	Date	Signature
Name of person taking consent	Date	Signature
Name of Doctor confirming eligibility for randomisation	Date	Signature
